# ELECtric Tibial nerve stimulation to Reduce Incontinence in Care homes: protocol for the ELECTRIC randomised trial

**DOI:** 10.1186/s13063-019-3723-7

**Published:** 2019-12-16

**Authors:** J. Booth, L. Aucott, S. Cotton, C. Goodman, S. Hagen, D. Harari, M. Lawrence, A. Lowndes, L. Macaulay, G. MacLennan, H. Mason, D. McClurg, J. Norrie, C. Norton, C. O’Dolan, D. A. Skelton, C. Surr, S. Treweek

**Affiliations:** 1 School of Health and Life Sciences, Glasgow Caledonian University, Govan Mbeki Building, Glasgow, G4 0BA UK; 2 Centre for Healthcare Randomised Trials (CHaRT), University of Aberdeen, Aberdeen, UK; 3 Centre for Research in Primary and Community Care, University of Hertfordshire, Hatfield, UK; 4 Nursing, Midwifery and Allied Health Professions Research Unit (NMAHP RU), Glasgow Caledonian University, Glasgow, UK; 5 Guy’s and St Thomas’ NHS Foundation Trust, London, UK; 6 Playlist for Life, Unit 1/14, Govanhill Workspace, Glasgow,, UK; 7 Health Services Research Unit, University of Aberdeen, Aberdeen, UK; 8 Yunus Centre for Social Business and Health, Glasgow Caledonian University, Glasgow, UK; 9 Usher Institute, Edinburgh University, Edinburgh, UK; 10 King’s College London, London, UK; 11 School of Health and Community Studies, Leeds Beckett University, Leeds, UK

**Keywords:** Care homes, Nursing home, Urinary incontinence, Tibial nerve stimulation, Older adults

## Abstract

**Background:**

Urinary incontinence (UI) is highly prevalent in nursing and residential care homes (CHs) and profoundly impacts on residents’ dignity and quality of life. CHs predominantly use absorbent pads to contain UI rather than actively treat the condition. Transcutaneous posterior tibial nerve stimulation (TPTNS) is a non-invasive, safe and low-cost intervention with demonstrated effectiveness for reducing UI in adults. However, the effectiveness of TPTNS to treat UI in older adults living in CHs is not known. The ELECTRIC trial aims to establish if a programme of TPTNS is a clinically effective treatment for UI in CH residents and investigate the associated costs and consequences.

**Methods:**

This is a pragmatic, multicentre, placebo-controlled, randomised parallel-group trial comparing the effectiveness of TPTNS (target *n* = 250) with sham stimulation (target *n* = 250) in reducing volume of UI in CH residents. CH residents (men and women) with self- or staff-reported UI of more than once per week are eligible to take part, including those with cognitive impairment. Outcomes will be measured at 6, 12 and 18 weeks post randomisation using the following measures: 24-h Pad Weight Tests, post void residual urine (bladder scans), Patient Perception of Bladder Condition, Minnesota Toileting Skills Questionnaire and Dementia Quality of Life. Economic evaluation based on a bespoke Resource Use Questionnaire will assess the costs of providing a programme of TPTNS. A concurrent process evaluation will investigate fidelity to the intervention and influencing factors, and qualitative interviews will explore the experiences of TPTNS from the perspective of CH residents, family members, CH staff and managers.

**Discussion:**

TPTNS is a non-invasive intervention that has demonstrated effectiveness in reducing UI in adults. The ELECTRIC trial will involve CH staff delivering TPTNS to residents and establish whether TPTNS is more effective than sham stimulation for reducing the volume of UI in CH residents. Should TPTNS be shown to be an effective and acceptable treatment for UI in older adults in CHs, it will provide a safe, low-cost and dignified alternative to the current standard approach of containment and medication.

**Trial registration:**

ClinicalTrials.gov, NCT03248362. Registered on 14 August 2017.

ISRCTN, ISRCTN98415244. Registered on 25 April 2018. https://www.isrctn.com/.

## Introduction

### Background and rationale

The highest prevalence of urinary incontinence (UI), defined by the International Continence Society as ‘any involuntary loss of urine’ [1], is found in residential or nursing care homes (CHs). UI is distressing for older adults and profoundly impacts on dignity and quality of life [2]. It is associated with impaired physical functioning [3], cognitive impairment [3, 4], sleep disturbance [2], falls [5, 6], fractures [7], urinary tract infection (UTI) [8] and hygiene and tissue viability problems [9]. UI affects social participation and is a major cause of clinical depression and social isolation [10, 11]. UI is costly to CH providers, healthcare services and the individual older adult. Direct personal and treatment costs are high. Intangible costs associated with social isolation and withdrawal from community participation also occur [10] but have not been quantified.

The most common type of UI experienced by older CH residents is mixed UI, combining symptoms of overactive bladder (OAB): urgency, frequency, nocturia with or without urgency UI, with stress UI [12]. For most this is exacerbated by functional losses of urine associated with frailty [13]. No evidence of the effects of conservative interventions directly addressing mixed incontinence in CH populations is yet available [12]. There is also a dearth of published evidence on interventions to promote recovery of bladder continence in the CH context [14] or for people living with dementia, even though the CH population is three times more likely to have UI or faecal incontinence (FI) than people of equivalent age and characteristics [15]. The burden of UI in the CH population is significant and increasing [3], yet evidence suggests that even intractable UI is amenable to interventions that may improve urinary function and quality of life [16]. Currently CHs use containment approaches, predominantly absorbent pads, rather than active treatment as the mainstay for managing UI [8]. Other non-pharmacological options include bladder training [17] and pelvic floor muscle training [18] as well as toileting programmes, signage and environmental adaptations for people with dementia. However, evidence indicates these are rarely used, have limited effectiveness in the CH environment and are labour intensive [19], which impacts on sustainability in the longer term. They also require a degree of cooperation, engagement and activity by the resident, which can be prohibitive for people with cognitive impairment [14, 20]. Antimuscarinic drugs may be used to reduce urge/OAB problems; however, these drugs are associated with significant adverse effects in frail older people and should be avoided in those with dementia, as they may also counteract the functional benefits of anticholinesterase inhibitors [21]. Newer β_3_-adrenergic receptor agonist drugs with potential benefit such as mirabegron are available, but the frail CH population has not been included in drug trials and, with prevalent polypharmacy in such contexts, any additional medication may increase adverse effects [22].

Transcutaneous posterior tibial nerve stimulation (TPTNS) is a simple, non-invasive, safe and low-cost intervention with promising effectiveness, directly targeting urgency or mixed UI [23, 24]. It uses a portable transcutaneous electrical nerve stimulation (TENS) machine to stimulate the posterior tibial nerve using surface electrodes placed adjacent to the medial malleolus. It does not require the resident to actively engage in order to receive the intervention, and so is suitable for those who are physically and cognitively frail. It is comfortable to use [25] and promotes dignified care, as only access to the resident’s ankle is required. It has been shown in randomised controlled trials (RCTs) to reduce UI in community-living older women [24] and adults with neurogenic bladder dysfunction (including multiple sclerosis [26], Parkinson’s [27] and stroke [28]); however, no definitive RCTs have focused on treating UI in the CH population. A small randomised feasibility study indicated the safety, acceptability and potential effects of TPTNS in this context [23].

Although the exact mechanism of action has yet to be fully understood, TPTNS is believed to restore the balance between excitatory and inhibitory bladder functioning by modulating the signal traffic to and from the bladder through the sacral plexus [29]. It is hypothesised that stimulating afferent sacral nerves in the lower extremities increases the inhibitory stimuli to the efferent pelvic nerve, suppresses bladder afferent nerve activity, reduces detrusor contractility and increases bladder capacity [30], and by these means TPTNS reduces the sensation of urgency and the frequency of micturition, thus enabling improved bladder control. These mechanisms may also reduce the volume of urine retained in the bladder after voiding [23, 26]. For the CH residents who wear absorbent pads because of mixed/urgency UI, TPTNS may reduce the sudden urge to urinate and frequency of voiding, allowing residents more time to reach the toilet, which in turn will enable more appropriate use of the toilet, generating respect and enhancing the person’s dignity.

As a potentially therapeutic modality, TPTNS could occupy a unique position in the CH care pathway for UI, as it provides active treatment of the mixed/urgency UI condition without requiring any active contribution by the resident. Thus, unusually, it is as likely to be of benefit to those with cognitive impairment as those without, and has been shown to be safe and not associated with any severe or limiting adverse effects. Skin redness and potential skin allergy are the only mild adverse effects reported.

A systematic review of TPTNS for UI identified ten RCTs [31]. A total of 472 participants were included, only 30 of whom were from a single CH population. All studies reported improvements in bladder condition with TPTNS, in terms of symptom improvement and/or UI-related quality of life, although no one trial was definitive. A meta-analysis (two trials) found a mean difference between TPTNS and control group in the self-reported International Consultation on Incontinence Questionnaire-Urinary Incontinence (ICIQ-UI) short-form score of − 3.79 (95% confidence interval [CI] – 5.82, – 1.76), which was considered a clinically meaningful effect [32]. There were no significant adverse events (AEs), and TPTNS was consistently reported as safe. However, the studies in the meta-analysis were small (outcomes from 79 participants), with methodological weaknesses and a Grading of Recommendations Assessment, Development and Evaluation (GRADE) [33] rating of ‘low quality’. With the increases in the older adult population and concomitant multimorbidities including dementia [16], together with the associated increase in numbers with UI, especially OAB/urgency incontinence [3, 12], there is a pressing need to investigate interventions to treat UI to reduce the burden on CH residents and care providers.

### Objectives

The ELECTRIC trial will:

1. Establish whether TPTNS is more effective than sham stimulation for reducing the volume of UI at 6, 12 and 18 weeks, in CH residents
2. Investigate mediating factors that impact on the effectiveness of TPTNS in a mixed method, process evaluation involving fidelity assessment, implementation support and qualitative components
3. Undertake economic evaluation of TPTNS in CHs assessing the costs of providing the programme and presenting the findings alongside the key primary and secondary outcomes in a cost consequence analysis
4. Explore in an interview study the experiences of TPTNS from the perspectives of:
  CH residents
  Family carers
  CH nurses and senior carers
  CH managers.

### Trial design

The research comprises a pragmatic, multicentre, placebo-controlled randomised parallel-group trial to compare the effectiveness of TPTNS (target *n* = 250) with sham stimulation (target *n* = 250) to reduce volume of UI in CH residents. Results from an internal pilot with 100–140 residents will determine progression to the main trial. A longitudinal, mixed methods nested process evaluation will run in parallel with the RCT to investigate intervention fidelity and acceptability, as well as qualitative exploration of the intervention delivery and implementation support. An economic evaluation of TPTNS compared with usual continence care will be completed in the form of a cost consequence analysis. The trial is designed in accordance with the Standard Protocol Items: Recommendations for Interventional Trials (SPIRIT) checklist (see Additional file 3).

## Methods: participants, interventions and outcomes

### Study setting

The settings will be CHs (nursing or residential) for older adults in England and Scotland.

### Eligibility criteria

The inclusion criteria are described as follows. CH residents will be eligible for inclusion if they have self- or staff-reported UI more than once per week; if they use the toilet or a toilet aid for bladder emptying with or without assistance; and if they wear absorbent pads to contain urine.

Exclusion criteria include CH residents who:

1. Have an indwelling urinary catheter
2. Have symptomatic UTI
3. Have post void residual urine (PVRU) volume more than 300 ml
4. Have a cardiac pacemaker
5. Have treated epilepsy
6. Have bilateral leg ulcers
7. Have pelvic cancer (current)
8. Are in palliative care status
9. Are non-English speakers.

### Who will take informed consent?

Processes for identifying eligibility and participant recruitment will differ in England and Scotland according to relevant legislation on capacity to provide informed consent to participate. In both countries the local Principal Investigator (PI, senior clinical nurse or manager) in each CH will identify potentially eligible residents, provide study information to all those with capacity and seek agreement from the residents for them to be approached by the Regional Research Assistant (RRA-registered nurses working in trial regions in Scotland and England) to receive further information about the study. All resident recruitment will be undertaken by the RRAs. In CHs in England in accordance with the Mental Capacity Act 2005 [34], where the local PI believes a resident’s capacity is in question, they will identify and provide the information to the resident’s personal consultee (usually a family member or friend), or if one is not available, a nominated consultee identified by the CH study team, and seek their agreement for an approach from the RRA. The RRA will provide a full explanation of the study, ensure eligibility and seek the consultee’s advice on what they feel the resident’s wishes would be about taking part in the trial, if they had capacity. The consultee will sign a declaration form if they believe the resident would choose to agree to participate. In CHs in Scotland, in accordance with the Adults with Incapacity (Scotland) Act 2000 [35], where a resident has a certificate of incapacity the local PI will identify and provide the study information to the resident’s welfare attorney (if one has been appointed) or their nearest relative. If there is no welfare attorney identified, or the resident does not have a relative who can be consulted, they will be considered ineligible to participate in the study. The local PI will seek agreement from the welfare attorney/nearest relative for the RRA to speak to them. The RRA will provide a full explanation of the study to the welfare attorney/nearest relative, ensure eligibility and seek written consent for the resident to participate. Written consent to participate in the process evaluation interviews will be sought by the RRA from the individual family carers or CH staff prior to the resident taking part.

### Additional consent provisions for collection and use of participant data and biological specimens

On the consent form, participants will be asked if they agree to use of their data should they choose to withdraw from the trial. Participants will also be asked for permission for the research team to share relevant data with people from the universities taking part in the research or from regulatory authorities, where relevant. This trial does not involve collecting biological specimens for storage.

## Interventions

### Explanation for the choice of comparators

To ensure the resident and their relatives are blind to the allocated intervention group, a sham stimulation intervention rather than a no-treatment comparator will be used. The sham stimulation will comprise low-intensity, subclinical stimulation of the lateral submalleolar area, positioned specifically on the lateral aspect of the ankle in order to avoid the tibial nerve, which runs close to the skin surface behind the medial malleolus. The cathode electrode will be positioned behind the lateral malleolus and the anode 10 cm cephalad to it. The stimulation parameters will be identical to the TPTNS stimulation other than the intensity of the current, which will always be set at 4 mA, not adjusted to individual comfort levels as it is in the TPTNS intervention group. The current will be initially increased until the resident reports feeling some sensation, following which the current will be reduced to 4 mA. All residents will be informed that they may not feel anything with this intervention and that this is quite normal. A previous pilot study [23] found that older residents were unable to accurately identify their allocated group and confirmed the integrity of the sham stimulation protocol.

### Intervention description

TPTNS is a form of peripheral neuromodulation. The tibial nerve, which lies immediately posterior to the medial malleolus, will be stimulated electrically using a portable TENS machine and two surface electrodes. The cathode electrode will be positioned behind the medial malleolus and the anode 10 cm cephalad to it. Standardised stimulation parameters will be applied of 10 Hz frequency, and a pulse width of 200 μs, in continuous stimulation mode. Intensity of stimulation (mA^-1^) will be adjusted on a session-by-session basis according to individual resident highest tolerated intensity below the motor threshold that remains comfortable. Both intervention and placebo/sham groups will receive an electrical stimulation programme comprising a total of 12 sessions of 30 min duration each, delivered twice weekly over 6 weeks. The stimulation equipment and method of delivery will be identical in everything but the intensity of electrical stimulation applied and the positioning of the surface electrodes. The electrical stimulators will be programmed to the set parameters and locked prior to individual use so that the only adjustable parameter will be the intensity of stimulation. The intervention will be delivered by CH registered nurses and senior carers who will receive specific training and support to undertake this role. No strict TPTNS/sham intervention timetable will be set, and individual CHs will have flexibility around where, how and when they deliver the sessions, bearing in mind that they should occur twice weekly for 30 min each, over a 6-week period. A proposed schedule for each home and resident will be agreed between the resident, registered nurse/senior carer and local PI at the point of treatment inception. The allocated treatment (TPTNS or sham) will be offered to the resident a maximum of two times in any 24-h period. If refused when first offered (verbally or by non-verbal behaviour), the treatment will be postponed for at least an hour and then offered one further time. Records of acceptance and refusals will be documented in the resident’s treatment diary. Adherence to the TPTNS or sham stimulation programme will be one of the progression criteria to full-scale trial from the internal pilot. While aiming to complete a full 12-session programme over the 6-week intervention period, contingency measures will be implemented if four or more sessions are refused or missed by the resident. Such measures will include approaching the resident at a later time, a different place or on a different day.

### Criteria for discontinuing or modifying allocated interventions

There will be no special criteria for discontinuing or modifying allocated interventions. Residents will remain in the trial unless they (or their welfare attorney/nearest relative in Scotland) choose to withdraw consent, or if their personal or nominated consultee (in England) advises that they believe the person’s wishes about participation have changed, or they are unable to continue for a clinical reason or if they die.

### Strategies to improve adherence to interventions

An individual resident stimulation diary will be completed by the registered nurse/senior carer following each session, recording date, time, intensity of electrical stimulation and any comments on the process of delivery. The locked stimulation machines will automatically record the total stimulation time in use and the average stimulation intensity of all recorded sessions, thus providing an objective record of the stimulation programme provided to each resident. This recorded data will be compared against the individually written stimulation diary completed by the staff after each session. The fidelity comparison will be performed by an Implementation Support Facilitator (ISF) whose role will be to provide support and ongoing training to CH staff and ensure they are competent and confident to deliver the stimulations. Electrode positioning, indicating accuracy of the allocated intervention, will be recorded using a digital photograph taken by staff every 2 weeks during the intervention delivery period, and will be viewed by the ISF.

### Relevant concomitant care permitted or prohibited during the trial

Implementing TPTNS or sham stimulation will not require alteration to current continence care pathways (including use of any medication), and these will continue in line with CH policies for both trial arms.

### Provisions for post trial care

Each participating CH will be provided with electrical stimulator machines after the trial follow-up period has been completed, should the homes wish to continue use of TPTNS.

## Outcomes

Table 1 summarises the outcomes assessed at baseline and at the 6 weeks, 12 weeks and 18 weeks post randomisation assessments.

**Table 1:** Outcomes assessed at trial time points

	Baseline	6-week	12-weeks	18weeks	Data collector
24 hour PWT^a^	●	●	●	●	CH staff and RRA
Number of pads used	●	●	●	●	RRA
72 hour bladder diary	●	●	●	●	CH staff
PVRU^b^	●	●	●	●	RRA
PPBC^c^	●	●	●	●	Resident and RRA
FC-PBC^d^	●	●	●	●	Family member
S-PBC^e^	●	●	●	●	SC/RN responsible for care provision
MTSQ^f^	●	●	●	●	Resident and RRA
MTSQ^f^	●	●	●	●	CH staff and RRA
DEMQOL^g^	●	●		●	Resident and RRA
DEMQOL-proxy^h^	●	●		●	Single, named proxy and RRA
Resource Use Questionnaire	●	●		●	RRA

^a^Pad Weight Test

^b^Post Void Residual Urine volume

^c^Patient Perception of Bladder Condition

^d^Family Carer Perception of Bladder Condition

^e^Staff Perception of Bladder Condition

^f^Minnesota Toileting Skills Questionnaire

^g^Measure of health-related quality of life in people with dementia (resident)

^h^Measure of health-related quality of life in people with dementia (proxy)

### Primary outcome measure

The primary outcome is the volume of urine leaked over a 24-h period at 6 weeks post randomisation.

### Secondary outcome measures

Secondary urinary outcome measures are as follows:

- Volume of urine leaked over a 24-h period at 12 and 18 weeks post randomisation
- Number of pads used in 24 h at 6, 12 and 18 weeks post randomisation
- PVRU at 6, 12 and 18 weeks post randomisation
- The Patient Perception of Bladder Condition [36] (PPBC) at 6, 12 and 18 weeks post randomisation
- The Minnesota Toileting Skills Questionnaire [37] (MTSQ) at 6, 12 and 18 weeks post randomisation.

### Quality of life outcomes

The quality of life outcome measures are:

- Resident Dementia Quality of Life (DEMQOL) [38] at 6 and 18 weeks post randomisation
- For those not able to complete it themselves, a proxy DEMQOL [38] at 6 and 18 weeks post randomisation.

### Economic outcomes

For economic outcomes, the Resource Use Questionnaire (RUQ) (Additional file 2) will be applied at 6 and 18 weeks post randomisation

## Participant timeline

**Fig. 1:**
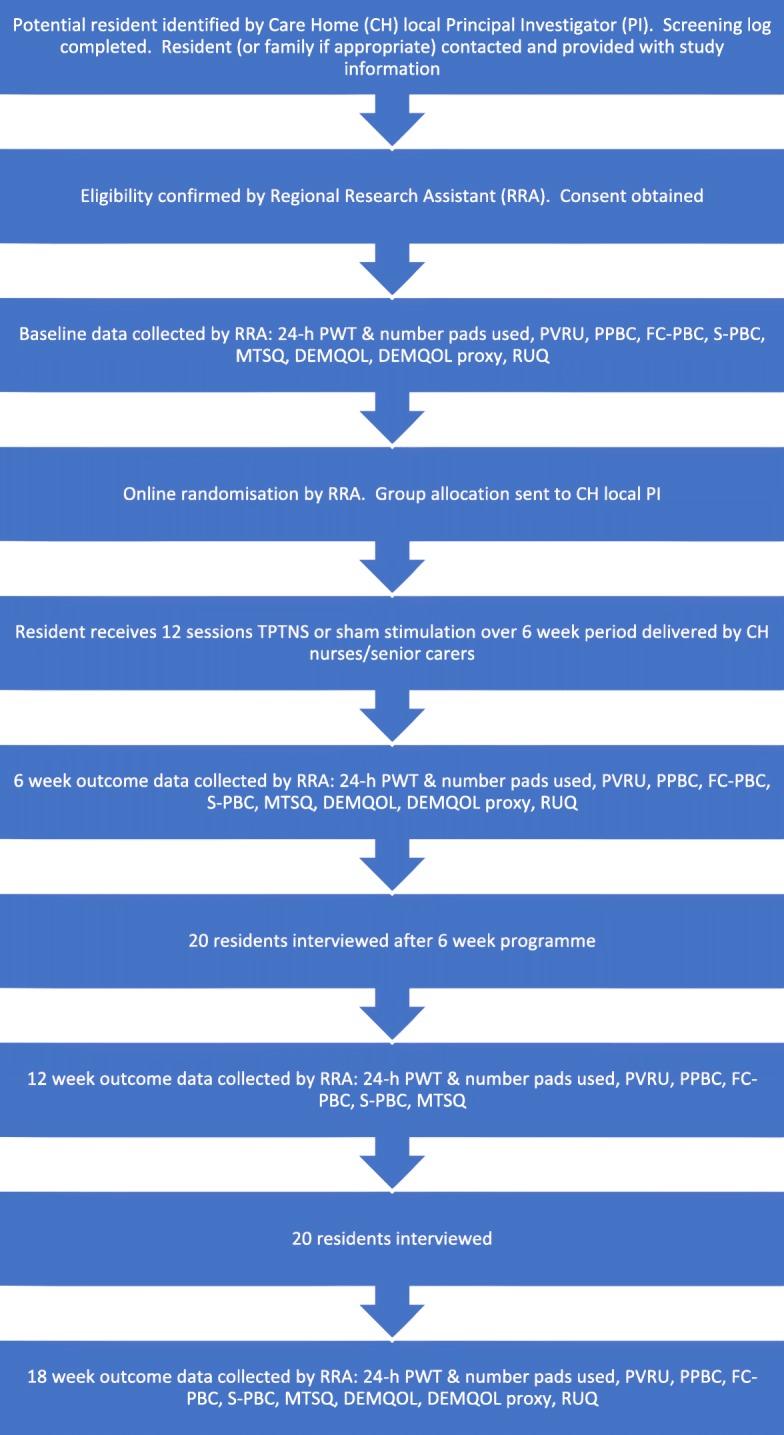
Resident flowchart

See Fig. 1 for the resident flowchart.

### Sample size

A total of 500 residents will be recruited. It was believed that the intervention may result in a worthwhile difference of a reduction of 200 ml/24 h in UI, equivalent to 200 g in pad weight [39]. There is a lack of data available on the standard deviation (SD) of Pad Weight Tests [40] (PWTs) in CH residents; however, a small RCT [39] reported results on this outcome. The SD from the trial was 450 ml, but given the small and selected sample included, the upper 95% CI around the SD was estimated and found to be approximately 570 ml/24 h, hence resulting in a standardised effect size of 0.35 (i.e. 200/570). To detect that difference with 90% power at the two-sided 5% alpha level, primary outcome data on 344 participants will be required. The limited data available suggested that the intra-class correlation (ICC) for any possible clustering effect was likely to be negligible. However, the sample size will be inflated to 500 to compensate for attrition in the primary outcome due to death of CH residents and potential transfers to other CHs, estimated conservatively to be 30% [40].

### Recruitment

CHs with a minimum of 25 residents will be eligible to take part. The minimum population of residents will be 1700, although the expected number will be considerably higher. Approximately 70% will have UI, and 10% will be in receipt of palliative care or do not use the toilet/toilet aid for elimination. Thus, there will be an estimated minimum total pool of 1071 eligible residents from whom 500 will be recruited. Assuming similar recruitment rates to the Health Technology Assessment (HTA)-funded Dementia Care Mapping (DCM)-EPIC study undertaken in CHs of 60% of eligible CH residents [41], this would provide at least 643 residents from whom to recruit a sample of 500. Recruitment will be undertaken over an 18-month period, commencing with month 6 and completed by month 24. At least four new CH sites will be established every 3 months during the recruitment period, depending on CH size and location.

## Assignment of interventions: allocation

### Sequence generation

Eligible, consenting residents are randomised to one of the two groups (TPTNS or sham). Randomisation is computer allocated on a one-to-one basis in random permuted blocks of size two, four or six, with stratification by:

- Sex (male/female)
- UI severity (mild [0–200 ml/24 h]; moderate [200–400 ml/24 h]; severe [400+ ml/24 h])
- Centre (CH).

### Concealment mechanism

Randomisation will utilise a proven web-based randomisation system, hosted by the Centre for Healthcare Randomised Trials (CHaRT) Unit, which ensures allocation concealment.

### Implementation of allocation sequence

The allocation sequence generation will be embedded in the trial website. After participants are enrolled, their baseline information will be entered onto the randomisation database remotely by the RRAs. The randomisation results will be automatically generated and emailed to the ELECTRIC trial office, who will forward them to the relevant local PI.

## Assignment of interventions: blinding

### Who will be blinded

Information required to perform the randomisation will be submitted by the RRA who obtained the consent. However, to ensure the RRA is blinded to group allocation, the information on the allocated group will be delivered to the local PI in each CH by the ELECTRIC trial office, who will receive the allocation information from CHaRT. The local PI will record the allocated group in a separate file and inform the CH staff who will deliver the allocated intervention.

### Procedure for unblinding if needed

We do not anticipate any requirement for unblinding, but if required, the Trial Manager, Data Coordinator, ISFs and CH managers will have access to group allocations, and any unblinding will be reported.

## Data collection and management

### Plans for assessment and collection of outcomes

Outcomes will be measured at 6, 12 and 18 weeks post randomisation. The primary outcome is the volume of urine leaked in 24-h at 6 weeks post randomisation, as measured by a 24-h PWT [40]. PWTs at 12 and 18 weeks measure the sustainability of any effect. The test is based on the premise that 1 g fluid weight = 1 ml urine and is thus an objective measure of urine leakage. The PWT involves the resident emptying their bladder, applying a clean, dry pad at an agreed set time and retaining all pads used between this time and 24 h later. To maintain the moisture in the removed pads and prevent evaporation, all collected pads will be individually sealed in a small plastic bag and then placed in a larger re-sealable bag, which is weighed onsite by the RRA after the 24-h collection ends. The dry weight of the equivalent pads to those collected will be deducted from the total weight to provide the 24-h volume of UI leaked. Secondary outcome measures will include the number of pads used in 24 h, which may be expected to reduce if TPTNS is effective in reducing volume of UI and will be reflected in the economic evaluation.

PVRU will be measured using a non-invasive portable ultrasound bladder scanner. A pilot study conducted with CH residents [23] suggested a potential mean decrease of 55 ml in PVRU following a TPTNS programme compared to the sham stimulation group. It is thus worth investigating whether this was an artefact, or whether TPTNS impacts on urinary retention in the frail older adult population. Additionally, it is important to ensure that any effect of TPTNS in reducing bladder leakage is not as a result of an increase in retained urine volume.

The PPBC [36] is a single-question global patient-reported outcome measure of perceived bladder condition with six possible responses, ranging from ‘My bladder condition does not cause me any problems at all’ to ‘My bladder condition causes me many severe problems’. It has good construct validity and responsiveness to change [36] and is recommended as a global outcome measure for UI [37]. It will be used at each time point with residents. However, it will also be adapted in this study for use by family carers as the Family Carer Perception of Bladder Condition (FC-PBC) and by the CH staff as the Staff Perception of Bladder Condition (S-PBC) to offer a perspective on how they believe the resident feels about their bladder condition.

The MTSQ [37] is a five-question patient-reported outcome measure of degree of difficulty on a scale of 0 to 4 for completing five tasks involved in toileting. Scores range from 0 to 20, with higher scores indicating more difficulty. The MTSQ is a reliable and valid interviewer-administered measure of toileting skills in physically frail older women [37]. It will be completed at all time points by the resident and/or staff member.

Quality of life is measured using the DEMQOL and DEMQOL-Proxy [38], valid and reliable measures of health-related quality of life in people with dementia. The DEMQOL-Proxy will be completed by a single identified proxy for the resident. Both measures will be completed at the primary outcome point (6 weeks post randomisation) and at the 18 weeks follow-up assessment.

Economic evaluation will be undertaken using routine data available in CHs as well as information from the RUQ designed for this study (Additional file 2). The RUQ will be administered by the RRA, who will record at baseline the usual continence care pathway including details on usage of pads and other equipment, medication which may affect continence level and (if appropriate) number of staff required to assist residents to use the toilet. At the primary outcome point (6 weeks) and at the 18 weeks follow-up time point, the RRA will use the RUQ, in combination with the 24-h bladder diary, to update the continence care pathway. If residents have required any care from health professionals external to the CH as a result of their UI, this is also recorded on the RUQ. Staff time required for training and the delivery of the intervention will be recorded by each CH, including the number of hours and the staff grade.

#### Process evaluation

The longitudinal process evaluation will be undertaken concurrently with the RCT and will primarily involve undertaking qualitative interviews with a range of informants (Table 2). The objectives will be to explore the experiences of the TPTNS intervention from the perspectives of residents, family carers and CH staff and to explore factors affecting intervention implementation in the CH context and optimisation for sustainability. Additionally, data on stimulation time and intensity with individual residents will be automatically recorded by the stimulation machines, which will be allocated for use by a single resident only, in order to ensure accurate information on stimulation is collected. This objective information will be compared with diary information completed by staff.

**Table 2:** Process evaluation data collection

Process evaluation data collection	Focus of questioning/data collection
Resident (with and without capacity) and/or family carer interviews at 6 weeks (*n* = 20)	Experiences, impact and acceptability
Resident (with and without capacity) and/or family carer interviews at 12 weeks (*n* < 20)	Experiences of incontinence, impact and acceptability of TPTNS
CH nurses/senior carer focus group interviews at 4–6 months (*n* = 20, number of participants 60–100)	Organisation and care home provision of continence care and influencing factors
CH nurses/senior carer individual interviews at 4–6 months (*n* < 20)	Organisation and care home provision of continence care and influencing factors
CH managers individual telephone interviews (*n* = 20)	Care home culture, management values, perceived impact of continence intervention at the organisational level, economic effects. Strategic considerations for implementation, rollout and sustainability
Fidelity to group allocation monitoring	Digital photographs of electrode position and stimulation diaries completed by staff
Adherence to stimulation programme	Objective recording of stimulation time and average intensity and stimulation diaries completed by staff
24-h bladder diaries	Patterns of voiding and toilet use

**Table 3:** Administrative information

Trial registration with registry that adheres to World Health Organization trial registration data set	ISRCTN98415244 NCT03248362 (Clinical trial.gov number)
Protocol version	Version 2.0; 27.08.18
Funding	National Institute for Health Research, Health Technology Assessment programme, project number HTA 15/130/73
Name and contact information for the trial Sponsor	Professor Kay Currie, Associate Dean, Research and Professor of Nursing, School of Health and Life Sciences, Glasgow Caledonian University, Cowcaddens Road, Glasgow, G4 0BA, UK
Role of Sponsor	The Sponsor played no part in study design; collection, management, analysis, and interpretation of data; writing of the report; or the decision to submit the report for publication

#### Qualitative data collection

Qualitative interviews will be undertaken by a research assistant skilled in the application of qualitative methods and will explore experiences of TPTNS or sham stimulation and any perceived impact on continence status and quality of life from the perspective of the CH residents and their family carers. Attention will be given to understanding the intervention acceptability in the short and longer term, especially in comparison to other UI management strategies they may be familiar with, and the identification of potential adherence moderating factors for future TPTNS delivery. All interviews will be digitally recorded and transcribed verbatim in preparation for analysis.

*For resident and/or family carer interviews*, face-to-face semi-structured interviews will be undertaken with residents and/or family carers, either as individual interviews or dyads. A total of 20 interviews will be carried out at 6 weeks, on completion of the intervention. A maximum of 20 further interviews with different residents/carers will take place at the 12-week juncture. Purposive sampling of resident/carers for the qualitative interviews will be undertaken on the basis of maximum variability sampling [42] with regard to gender, age, bladder symptoms, cognitive and functional status and resident or carer status. Three quarters of the interviews will involve residents who have received the TPTNS intervention or their families. A topic guide for the semi-structured approach will be developed to ensure all questions of interest are addressed. Fewer interviews will be conducted if data saturation is reached.

*For CH nurses/senior carer interviews*, focus group (or small group) interviews will be undertaken with CH nurses and senior carers involved in the direct delivery of the TPTNS/sham intervention. Attention will be paid to understanding the organisation of care, how management works with care staff, level of staff turnover in the previous 6 months and how continence care is organised within the routines of the CH. One focus group per CH or, where for staffing reasons this is not possible to organise, two to three small group interviews, will be held during the month following the intervention completion. This will result in the equivalent of 20 focus group interviews involving 60–100 CH staff. Additionally, up to 20 individual interviews will be undertaken with nurses/senior carers delivering the intervention, to explore and elicit views which staff may be reluctant to share in a group interview.

*Regarding the CH managers*, individual telephone interviews with them (*n* = 20) will be completed at the end of each CH’s involvement with the study (6 months following site inception). The focus of these interviews will be to explore the CH culture and management values as well as perceived effects of the continence intervention at the organisational level, including any impact on culture and quality of care and any economic effects. Strategic considerations for implementation rollout and sustainability in the event that TPTNS is found to be effective will be identified and explored in depth.

### Plans to promote participant retention and complete follow-up

The CH resident population is relatively stable, with changes being largely the result of individual illness or death; therefore, discontinuation or change of status is anticipated rather than loss to follow-up. The collection of outcomes will be undertaken by a single RRA in each CH, who will be well known to the CH staff, and it is anticipated this design will support good retention and follow-up rates.

### Data management

Both paper-based and electronic data entry will be used. Data will be collected locally by the RRA and entered onto the database for screening and randomisation purposes. Paper-based Case Report Form (CRF) data will be delivered securely to the trial office for data entry.

### Confidentiality

All collected information will be kept strictly confidential and will be stored in accordance with the UK Data Protection Act 2018 [43] and retained in accordance with the latest Directive on Good Clinical Practice (GCP) and local policy. Data collected during the course of the research will be kept strictly confidential and only accessed by members of the trial team (or individuals from the Sponsor organisation or CH sites where relevant to the trial). Participants will be allocated an individual trial identification number. Participants’ details will be stored on a secure database under the guidelines of the 2018 General Data Protection Regulation (EU) 2016/679 [44]. The CHaRT senior IT manager (in collaboration with the Chief Investigator [CI]) will manage access rights to the data set. It is anticipated that anonymised trial data may be shared with other researchers to enable international prospective meta-analyses.

### Plans for collection, laboratory evaluation and storage of biological specimens for genetic or molecular analysis in this trial/future use

This aspect is not applicable; there are no biological specimens.

### Access to data

Data may be available for collaborators on request to the CI.

## Analysis

### Statistical methods for primary and secondary outcomes

All analyses will be undertaken according to a previously agreed statistical analysis plan (SAP), which will be agreed with the Trial Steering Group (TSC), including the independent statistician, before the database is locked and any data analysis commences.

#### Main effectiveness analysis

All baseline characteristics, follow-up measurements and safety data will be described using the appropriate descriptive summary measures: mean and SD for continuous and count outcomes or medians and inter-quartile range if required for skewed data, numbers and percentages for dichotomous or categorical outcomes. The primary outcome, measured at 6 weeks post randomisation, will be analysed using linear multivariable regression correcting for baseline 24- h PWT, the stratification design variables and other prognostic variables; all models will include a random effect for CH. The statistical analysis of the primary outcome will be by intention-to-treat (ITT); the effects of compliance with treatment will be explored using causal models to examine if the allocation to treatment impacts on participant adherence and fidelity fitting of the electrodes. Secondary outcomes will be analysed using a similar strategy employing generalised linear models suitable for the outcome. All treatment effects will be derived from these models and presented with 95% CIs. All analyses will be performed and reported in accordance with the Consolidated Standards of Reporting Trials (CONSORT) statement and the ICH E9 ‘Statistical Principles in Clinical Trials’ [45]. The main analysis will be performed at the end of the trial when the 18 weeks follow-up has been completed.

#### Qualitative research analysis

For the three sets of qualitative interview data, separate Framework analyses will be undertaken with the support of QSR NVivo (version 10) data management and analysis software. This method permits identification and cross-classification of variables directly from digital transcriptions. The analytic process will consist of identifying key concepts and themes and mapping their range and diversity, followed by a process of interpretation where patterns of association will be investigated and possible reasons for these explored. In achieving this, all transcripts will be summarised, charted and coded for recurrent themes. Specific analytic intentions are associated with each of the three interview data sets.

*For residents/family carer interviews*, the framework will be developed to explore the elements of perceived impact and acceptability of TPTNS as a therapeutic intervention, by residents and family carers, in both the short-term and for the longer-term.

*For the CH staff*, the focus group and individual interview framework will highlight the experience of CH staff in developing their new skill sets and the facilitators and challenges they experience implementing them into routine practice. The elements of the Capability, Opportunity, Motivation and Behaviour (COM-B) model which formed the theoretical underpinning of the staff interview schedule will be key concepts in this framework.

*For the CH managers*, the focus of the framework for analysing the CH managers’ interviews will be the cultural, economic, strategic and quality impacts associated with participating in the trial and implications for implementation and sustainability at the organisational level.

The coherence, transparency and validity of the interpretations from these three different framework analyses will be assessed through regular iterative discussion between the Research Assistant with qualitative research experience and the study team members with qualitative expertise.

### Interim analyses

An independent Data Monitoring and Ethics Committee (DMEC) will review confidential interim analyses of accumulating data at its discretion, but at least annually. There are no formal stopping rules.

### Methods for additional analyses (e.g. subgroup analyses)

#### Planned subgroup analyses

Subgroup analyses will be carried out accordingly by:

- Gender
- UI severity
- Dependency in toilet use
- Cognitive status
- Falls status.

The threshold for statistical significance for the subgroup analyses will be 0.01, reflecting the number of subgroup comparisons being made. Heterogeneity of treatment effects amongst subgroups will be tested for using the appropriate subgroup by treatment group interactions.

#### Process evaluation data analysis

Process evaluation data analysis will address adherence to the stimulation programme by group, at 6 weeks post randomisation, the end of the stimulation programme. Characteristics of residents and the stimulation programme received will be described using appropriate summary measures, and the proportion who received the therapeutic minimum (> 8 stimulation sessions) and the full 12-session programme will be presented. Overall fidelity to the allocated group will also be assessed and presented to illuminate resident elements of the outcome analysis, including total stimulation time, mean intensity of stimulation and accuracy of electrodeposition. Stimulation diaries will be analysed to inform understanding of when, how and who delivers the electrical stimulation in practice.

#### Economic evaluation

The economic evaluation will compare the costs and outcomes of TPTNS compared with usual continence care pathways and present these in a cost consequence analysis. Unit costs will be attached to the individual resources identified in the RUQ (Additional file 2) using standard sources (including National Health Service [NHS] Reference Costs, Unit Costs of Health and Social Care and British National Formulary [37, 46, 47]). Staff training time will be costed using the appropriate pay scales for each site. The costs of the trainer and the materials (TPTNS machines, handbook and training DVD) will be based on the market rates for these items. Data on costs will not be combined directly with the primary study outcome, as it is likely that this will not provide a representative reflection of the impact of the TPTNS intervention on this older group of CH residents. Relevant outcomes, including the trial primary and secondary outcomes, along with important issues from the process evaluation will be presented alongside the costs in a cost consequence analysis. Here the costs will be presented alongside the effects, both quantitative and qualitative, in a disaggregated format to allow flexibility in presenting which costs/effects are relevant to different stakeholders.

### Methods in analysis to handle protocol non-adherence and any statistical methods to handle missing data

Analysis will be by the ITT method. It is not currently planned to impute missing values, but multiple imputation or other strategies within sensitivity analysis may be considered (see the section on Statistical methods for primary and secondary outcomes). These will be pre-specified in the SAP.

### Plans to give access to the full protocol, participant-level data and statistical code

The full current protocol (version 2.0) is provided as Additional file 1 to this document. Anyone interested in other data or documentation should contact the corresponding author.

## Oversight and monitoring

### Composition of the coordinating centre and Trial Steering Committee

The trial office will be based in the School of Health and Life Sciences, Glasgow Caledonian University, providing day-to-day support for the trial. The local PI and RRA in each site will be responsible for all aspects of local organisation, including identifying potential recruits and taking consent. The trial will be supervised by the Project Management Group (PMG), which will meet every 3 months. The PMG will comprise grant holders and representatives from the trial office and CHaRT. A Trial Steering Committee (TSC), with six independent members, will meet four times over the course of the trial to oversee conduct and progress. A Stakeholder and Public Involvement Group (SPIG) will meet every 6 to 9 months to advise on trial processes and acceptability, and also to support interpretation and dissemination of findings.

### Composition of the data monitoring committee, its role and reporting structure

A DMEC will oversee the safety of subjects in the trial. The committee will meet regularly to monitor the trial data and make recommendations as to any required modifications to the protocol or the termination of all or part of the trial.

### Adverse event reporting and harms

In this trial, all adverse events (AEs) or serious adverse events (SAEs) occurring during an electrical stimulation (treatment/sham) session, or while equipment is attached to the resident’s leg, or during data collection periods will be recorded. Given the previous established safety profile of TPTNS, SAEs are not anticipated. In this trial the following related minor AEs are potentially expected:

- Transient skin redness at electrode sites
- Minor itch at electrode sites.

All AEs and SAEs will be assessed for expectedness, seriousness, severity and causality and will be reported to the DMEC and relevant regulatory bodies as required.

### Frequency and plans for auditing trial conduct

Three monthly PMG meetings facilitate review of trial conduct. The TSC and DMEC will also meet to review conduct throughout the trial period (four times and three times respectively).

### Plans for communicating important protocol amendments to relevant parties (e.g. trial participants, ethical committees)

The Investigators will conduct the trial in compliance with the protocol given favourable opinion by the Research Ethics Committee(s) (RECs). Any amendment to the trial will be approved by the Sponsor and funder before application to the RECs, except in the case of immediate safety measures, when the Sponsor will be notified as soon as possible. Any deviations from the protocol will be fully documented using a breach report form. The CHs will be notified of any protocol amendments, and a copy of the revised protocol will be sent to the PI to add to the Investigator Site File.

### Dissemination plans

The authors plan to publish the findings in a range of practice-focused journals and publications and make use of social media to enable rapid dissemination of not only the research results but also information on training and implementing TPTNS into practice, if the results indicate it is effective. Short, plain English summaries will be prepared to disseminate the findings to user groups and members of the public through websites, newsletters and social media.

## Discussion

UI is highly prevalent in residential and nursing CHs and has a profound impact on dignity and quality of life [2]. Currently, CHs use a containment approach to managing UI, predominantly using absorbent pads [8]. These are not only uncomfortable and undignified for the individual but are costly to the CH and health service providers. Anticholinergic drugs can also be used to treat UI, but they may have significant adverse effects in older adults and can adversely interact with drugs used to treat dementia [21].

TPTNS is a non-invasive intervention that has demonstrated effectiveness at reducing UI in adults [23]. However, there is a lack of evidence-based research into the safety, acceptability and effectiveness of its use in frail older adults. The ELECTRIC trial will test the feasibility and effectiveness of CH staff delivering TPTNS to adults in CHs. Economic evaluation will assess the costs of providing a programme of TPTNS, and the process evaluation will provide valuable information on the experiences of TPTNS from the point of view of CH residents, family members, CH staff and managers.

Should TPTNS be shown to be an effective and acceptable treatment for UI in older adults in CHs, it will provide a safe, low-cost and dignified alternative to the current standard approach of containment and medication.

### Trial status

The ELECTRIC trial is currently recruiting in five UK CHs and has completed recruitment in 38 homes. The first patient was randomised in February 2018, with current recruitment at 371 participants. Recruitment is due to be completed at the end of July 2019, and follow-up will be completed by the end of December 2019. The TSC and DMEC have convened three times. For updates see ClinicalTrials.gov and the ELECTRIC trial website [48]. The current version of the trial protocol (version 2.0) is provided in Additional file 1 and administrative information is provided in Table 3.

## Supplementary information

**Additional file 1.** Current version of trial protocol.

**Additional file 2.** Resource Use Questionnaire designed for the ELECTRIC trial.

**Additional file 3.** SPIRIT 2013 checklist: recommended items to address in a clinical trial protocol and related documents.

## Data Availability

Any data required to support the protocol can be supplied on request.

## Abbreviations

AE: Adverse event
AWI: Adults with Incapacity (Scotland) Act 2000
CH: Care home
CHaRT: Centre for Healthcare Randomised Trials
CI: Chief Investigator, confidence interval
CRF: Case Report Form
DEMQOL: Dementia Quality of Life
DMEC: Data Monitoring and Ethics Committee
FC-PBC: Family Carer Perception of Bladder Condition
FI: Faecal incontinence
GCP: Good Clinical Practice
GRADE: Grading of Recommendations Assessment, Development and Evaluation
HTA: Health Technology Assessment
ICIQ-UI: International Consultation on Incontinence Questionnaire-Urinary Incontinence
IRAS: Integrated Research Application System
ISF: Implementation Support Facilitator
ISRCTN: International Standard Randomised Controlled Trial Number
MCA: Mental Capacity Act 2005
MMSE: Mini Mental State Examination
MTSQ: Minnesota Toileting Skills Questionnaire
NHS: National Health Service
NIHR: National Institute for Health Research
NMAHP RU: Nursing, Midwifery and Allied Health Professions Research Unit
NRES: National Research Ethics Service
OAB: Overactive bladder
PI: Principal Investigator
PMG: Project Management Group
PPBC: Patient Perception of Bladder Condition
PVRU: Post void residual urine (volume)
PWT: Pad Weight Test
QALY: Quality-adjusted life year
QoL: Quality of life
RCT: Randomised controlled trial
REC: Research Ethics Committee
RN: Registered nurse
RRA: Regional Research Assistant
RUQ: Resource Use Questionnaire
SAE: Serious adverse event
SAP: Statistical analysis plan
SC: Senior carer
SD: Standard deviation
S-PBC: Staff Perception of Bladder Condition
TENS: Transcutaneous electrical nerve stimulation
TPTNS: Transcutaneous posterior tibial nerve stimulation
TSC: Trial Steering Committee
UI: Urinary incontinence
UK: United Kingdom
