# Supplementary material for: ELECtric Tibial nerve stimulation to Reduce Incontinence in Care homes: protocol for the ELECTRIC randomised trial
Source: Trials. 2019 Dec 16;20:723. doi: 10.1186/s13063-019-3723-7 (PMC6915984; doi:10.1186/s13063-019-3723-7)

**
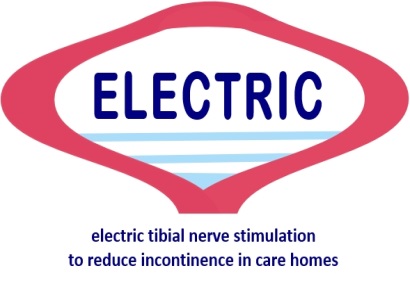
**

**ELECTRIC:**

**ELECtric Tibial nerve stimulation to**

**Reduce Incontinence in Care homes:**

**PROTOCOL**

A UK Collaborative Study funded by the

NIHR Evaluation, Trials and Studies Coordinating Centre (NETSCC),

Health Technology Assessment (HTA) Programme

**
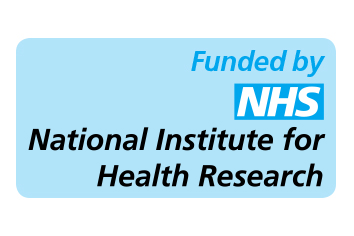
**

**
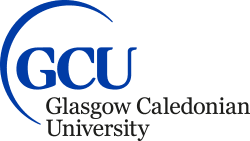
**

**Funding acknowledgement:** This research was funded by the NIHR HTA (15/130/73)

**Department of Health disclaimer:** The views expressed are those of the author(s) and not necessarily those of the NHS, the NIHR or the Department of Health

**Sponsor/Co-Sponsors** (add details of all Sponsors)

| Name: | Professor James Woodburn, |
| --- | --- |
| Address: | School of Health and Life Sciences, Glasgow Caledonian University, Cowcaddens Road, Glasgow, G4 0BA |
| Email: | Jim.woodburn@gcu.ac.uk |
|  |  |

**Chief-Investigator (CI)**

| Name: | Professor Joanne Booth |
| --- | --- |
| Address: | School of Health and Life Sciences, Glasgow Caledonian University, Cowcaddens Road, Glasgow, G4 0BA |
|  |  |
| Telephone: | +44 (0)1413318635 |
| E-mail: | Jo.booth@gcu.ac.uk |

**Trial Office**

| Address: | ELECTRIC Trial Office  3^rd^ Floor Govan Mbeki Building  Glasgow Caledonian University  Cowcaddens Road, Glasgow, G4 0BA |
| --- | --- |
| Telephone: | 0141 331 8106/3611 |
| Fax: | 0141 331 3005 |
| E-mail: | ELECTRIC@gcu.ac.uk |
| Website: | <http://www.charttrials.abdn.ac.uk/XXXXXX> - to be confirmed |

**Funder**

| Name: | NIHR Health Technology Assessment |
| --- | --- |
| Funder number: | HTA Project: 15/130/73 |
| Funder start date: | 1^st^ July 2017 |
| Funder end date: | 30 June 2020 |

**Other**

| Clinical trial.gov number | NCT03248362 |
| --- | --- |
| REC number (Scotland): | | 17/SS/0117 | | --- | |
| IRAS number: | IRAS Project ID 224515 |
| REC number (England) | | 17/YH/0328 | | --- | |
| IRAS number | IRAS Project ID 233879 |

**Signatures**

By signing this document I am confirming that I have read, understood and approve the protocol for the above trial.

| [Insert name of CI]: | Joanne Booth |
| --- | --- |
| Date: |  |

| [Insert name of Statistician]: | Dr Graeme Maclennan signature |
| --- | --- |
| Date: |  |

**VERSION HISTORY**

| **Amendment no.** | **Protocol version no.** | **Description of changes**  **(*incl. author(s) of changes*)** | **Date of protocol** |
| --- | --- | --- | --- |
|  | 0.1 | New Protocol | 24/04/17 |
|  | 0.11 | Addition of Minnesota Toilet Skills Questionnaire to information recorded in CRF at each measurement timepoint. – Added by Jo Booth | 16/07/17 |
|  | 0.12 | Removed repeat measures of Barthel Index, Mini Mental State Examination [MMSE], Clinical Frailty Scale [CFS], falls and fractures, treated UTIs, visits to accident and emergency in the previous 6 months, pressure ulcer risk and incidence in previous month – changes made by Jo Booth. | 10/08/17 |
|  | 0.13 | Acknowledgement section of the authorship agreements updated to consortium acknowledged in all publications – added by Catriona O’Dolan.  9.2 Replaced BUPA and ENRICH care homes with ‘large’ and ‘smaller’ to reflect wider site recruitment strategy – changes made by Jo Booth | 10/10/17 |
|  | 1.0 | REC request to add ‘non-English speaking’ to exclusion criteria.  Section 3.1 – Intervention: added detail about number of times in 24 hour period resident will be offered intervention and associated progression criterion from internal pilot to full trial. | 17/11/17 |
|  | 2.0 | Trial Personnel: change of university for TSC member Dr Chris Sutton; change of job title and university for Prof Simon Skene member of DMEC  Section 3.4 describing the internal pilot study added  Section 4.1 Changed 20 care homes to >20 care homes.  Section 4.4. Removed mention of BUPA Research Ethics and Approval processes.  Section 4.7 Removed paragraph on ‘ascertainment bias as 72-hour bladder diary replaced with 24 hour bladder diary. Replaced ‘local PI’ in paragraph on contamination with ‘Implementation Support Facilitator’  Section 6.1 table 1 Changed 72-hour bladder diary to 24-hour bladder diary  Section 6.2 and 6.3 Changed 72-hour bladder diary to 24-hour bladder diary  Safety section 7 updated to reflect SOP for adverse/serious adverse event reporting:  Section 7.2 Clarified meaning of AEs and SAEs in this trial  Section 7.3.2 Clarified reporting processes and responsibilities for AEs/SAEs  Section 7.3.3 Clarified responsibilities for determining seriousness and relatedness of AEs/SAEs  Section 9.1 Proportion of 500 residents recruited in two regions  Section 9.2 Number of large and small CHs removed. Minimum number of CH residents reduced to 25. Removal of fig 1 (recruitment schedule based on 20 homes)  Section 10.3 Process evaluation data analysis added  Section 11.1 3-day bladder diary changed to 24hr bladder diary  Section 12 Figure 2 changed to Figure 1  Section 13.2 Updated to GDPR, replacing Data Protection Act 1998  Appendix 2 20 CHs changed to >20 CHs  All protocol changes made by Jo Booth | 27/08/18 |
|  |  |  |  |

**TABLE OF CONTENTS**

[PROTOCOL SUMMARY 6](#__RefHeading___Toc479165838)

[GLOSSARY OF ABBREVIATIONS 7](#__RefHeading___Toc479165839)

[TRIAL PERSONNEL 8](#__RefHeading___Toc479165840)

[1. INTRODUCTION 10](#__RefHeading___Toc479165841)

[2. TRIAL AIM AND OBJECTIVES 11](#__RefHeading___Toc479165842)

[3. TRIAL DESIGN 12](#__RefHeading___Toc479165843)

[4. TRIAL RECRUITMENT 16](#__RefHeading___Toc479165844)

[5. OUTCOME MEASURES 19](#__RefHeading___Toc479165845)

[6. DATA COLLECTION AND PROCESSING 19](#__RefHeading___Toc479165846)

[7. SAFETY 23](#__RefHeading___Toc479165847)

[8. EMBEDDED QUALITATIVE WORK 24](#__RefHeading___Toc479165848)

[9. SAMPLE SIZE AND PROPOSED RECRUITMENT RATE 26](#__RefHeading___Toc479165849)

[10. STATISTICAL ANALYSIS 27](#__RefHeading___Toc479165850)

[11. ECONOMIC EVALUATION 28](#__RefHeading___Toc479165851)

[12. ORGANISATION: TRIAL MANAGEMENT AND OVERSIGHT ARRANGEMENTS 29](#__RefHeading___Toc479165852)

[13. RESEARCH GOVERNANCE, DATA PROTECTION AND SPONSORSHIP 30](#__RefHeading___Toc479165853)

[14. ETHICS AND REGULATORY APPROVALS 30](#__RefHeading___Toc479165854)

[15. QUALITY ASSURANCE 31](#__RefHeading___Toc479165855)

[16. FINANCE AND INSURANCE 31](#__RefHeading___Toc479165856)

[17. END OF TRIAL 31](#__RefHeading___Toc479165857)

[18. DATA HANDLING, RECORD KEEPING AND ARCHIVING 31](#__RefHeading___Toc479165858)

[19. SATELLITE STUDIES 31](#__RefHeading___Toc479165859)

[20. AUTHORSHIP AND PUBLICATION 32](#__RefHeading___Toc479165860)

21. REFERENCE LIST ..................................................................................................................... 29

[Appendix 1: Authorship Policy 32](#__RefHeading___Toc479165861)

Appendix 2: Study flowcharts

# PROTOCOL SUMMARY

| **Question addressed** | Is a programme of transcutaneous posterior tibial nerve stimulation (TPTNS) a clinically effective treatment for urinary incontinence (UI) in care home residents and what are the associated costs and consequences? | |
| --- | --- | --- |
|  |  | |
| **Considered for entry** | Men and women resident in care homes, with UI of at least weekly, including those with cognitive impairment. | |
|  |  | |
| **Inclusion/Exclusion criteria** | ***Inclusion criteria:*** Care home residents:  (i) with self or staff reported of weekly or more frequent UI  (ii) who use toilet/toilet aid to evacuate bladder  (iii) who wear absorbent pads to contain UI  ***Exclusion criteria:*** Care home residents  (i) with an indwelling urinary catheter  (ii) with symptomatic urinary tract infection  (iii) with post-void residual urine volume more than 300ml  (iv) with a cardiac pacemaker  (v) with treated epilepsy  (vi) with bilateral leg ulcers  (vii) with pelvic cancer  (viii) on the palliative care register  (ix) non-English speakers | |
|  |  | |
| **Interventions** | 1.Programme of transcutaneous posterior tibial nerve electrical stimulation | 2.Programme of sham stimulation |
|  |  | |
| **Outcomes** | Volume of UI over 24 hour period  Number of pads used in 24 hours  Post-void residual urine volume  Resident, family carers, staff perception of bladder condition  Resident Toileting Skills  Quality of life | |
|  |  | |
| **Co-ordination** | **Local**: by local lead Principal Investigator (Care Home Manager or Senior Clinical Nurse)  **Central**: by Trial Office in Glasgow  (Telephone 0141 331 8106/3611).  **Overall**: by the Project Management Group and overseen by the Trial Steering Committee and the Data Monitoring and Ethics Committee. | |

| GLOSSARY OF ABBREVIATIONS | |
| --- | --- |
| AE | Adverse Event |
| AWI | Adults with Incapacity (Scotland) Act 2000 |
| CH | Care Home |
| CHaRT | Centre for Healthcare Randomised Trials |
| CI | Chief Investigator |
| CONSORT | Consolidated Standards of Reporting Trials |
| CRF | Case Report Form |
| DMEC | Data Monitoring and Ethics Committee |
| FI | Faecal Incontinence |
| GCP | Good Clinical Practice |
| GP | General Practitioner |
| HSRU | Health Services Research Unit |
| HTA | Health Technology Assessment |
| ISF | Investigator Site File |
| ISRCTN | International Standard Randomised Controlled Trial Number |
| IVR | Interactive Voice Response (randomisation) |
| MCA | Mental Capacity Act 2005 |
| MMSE | Mini Mental State Examination |
| MTSQ | Minnesota Toileting Skills Questionnaire |
| NHS | National Health Service |
| NIHR | National Institute Health Research |
| NRES | National Research Ethics Service |
| OAB | Over Active Bladder |
| PI | Principal Investigator |
| PIL | Patient Information Leaflet |
| PMG | Project Management Group |
| PPI | Patient and Public Involvement |
| PTNS | Percutaneous Posterior Tibial Nerve Stimulation |
| PVRU | Post Void Residual Urine volume |
| PWT | Pad Weight Test |
| QoL | Quality of Life |
| RCT | Randomised Controlled Trial |
| REC | Research Ethics Committee |
| RN | Registered Nurse |
| RRA | Regional Research Assistant |
| RUQ | Resource Use Questionnaire |
| SAE | Serious Adverse Event |
| SAP | Statistical Analysis Plan |
| SC | Senior Carer |
| SD | Standard Deviation |
| SOP | Standard Operating Procedure |
| TENS | Transcutaneous Electrical Nerve Stimulation |
| TMF | Trial Master File |
| TPTNS | Transcutaneous Posterior Tibial Nerve Stimulation |
| TSC | Trial Steering Committee |
| UK | United Kingdom |
| UKCRC | United Kingdom Clinical Research Collaboration |
| UI | Urinary Incontinence |
| UoA | University of Aberdeen |

# TRIAL PERSONNEL

Chief Investigator

| 1 | Professor Joanne Booth |
| --- | --- |

Grant Holders

| 1 | Professor Suzanne Hagen | 9 | Professor John Norrie |
| --- | --- | --- | --- |
| 2 | Professor Doreen McClurg | 10 | Professor Shaun Treweek |
| 3 | Dr Maggie Lawrence | 11 | Dr Danielle Harari |
| 4 | Dr Helen Mason | 12 | Mr Andrew Lowndes |
| 5 | Professor Dawn Skelton | 13 | Mr Graeme MacLennan |
| 6 | Professor Christine Norton |  |  |
| 7 | Professor Claire Surr |  |  |
| 8 | Professor Claire Goodman |  |  |

**Trial Office Team - at Glasgow Caledonian University**

| 1 | Chief Investigator |  |  |
| --- | --- | --- | --- |
| 2 | Trial Manager |  |  |
| 3 | Data Co-ordinator |  |  |
| 4 | Qualitative research assistant |  |  |
| 5 | Regional Research assistant |  |  |
| 6 | Regional Research assistant |  |  |

**CHaRT team – at Aberdeen University**

| 1. CHaRT Director |  |  |
| --- | --- | --- |
| 1. Senior Trial Manager |  |  |
| 1. Trial statistician |  |  |
| 1. Senior IT Manager |  |  |
| 1. Programmer |  |  |
|  |  |  |

**Project Management Group (PMG)**

This Group is comprised of all grant holders along with representatives from the Trial Office team and CHaRT trial team.

**Trial Steering Committee (TSC) Members**

**The membership of this Committee comprises independent members along with the** Chief Investigator (Joanne Booth). The other ELECTRIC grant-holders and key members of the Trial Office team (e.g. the trial manager) may attend TSC meetings. The funders and sponsor will be notified in advance of meetings and a representative invited to attend. Other relevant experts may be invited to attend as appropriate.

**Independent TSC members:**

| 1 | Professor Pip Logan, University of Nottingham (Chair) |
| --- | --- |
| 2 | Dr Christopher Sutton, Statistician, University of Manchester |
| 3 | Dr Lois Thomas, Continence and Nursing expert, UCLAN |
| 4 | Prof Francine Cheater, Independent PPI representative and independent continence and nursing expert. |
| 5 | Mr Joby Taylor, Consultant Urological Surgeon, NHS Forth Valley. Independent urology expert |
| 6 | Mrs Joyce Goel, Independent PPI representative. |

**Data Monitoring Committee (DMEC) Members**

**This Committee comprises independent members, and the trial statistician contributes as appropriate.** The CI and/or a delegate may contribute to the open session of the meetings as appropriate.

| 1 | Dr Terence Quinn, Lecturer in Geriatric Medicine, University of Glasgow (Chair) |
| --- | --- |
| 2 | Dr Adam Gordon, Clinical Associate Professor in Medicine of Older People, University of Nottingham |
| 3 | Professor Simon Skene, Director of Surrey Clinical Trials Unit, University of Surrey |

**ELECtric Tibial nerve stimulation to Reduce Incontinence in Care homes: ELECTRIC**

## 1. INTRODUCTION

**1.1 Background**

The highest prevalence of urinary incontinence (UI), defined by the International Continence Society as ‘any urinary leakage’, is found in residential or nursing care homes (CH). UI is distressing for older adults and profoundly impacts on dignity and quality of life^6^. It is associated with impaired physical functioning^7^ cognitive impairment ^7,8^ sleep disturbance^6^, falls, fractures^9^, hygiene and tissue viability problems^10^. UI affects participation by older adults and is a major cause of clinical depression and social isolation^11,12,13^. Incontinence is costly, to CH providers, the NHS and the individual older adult. Direct personal and treatment costs are high. Intangible costs associated with social isolation and withdrawal from participatory groups also occur but have not been quantified.

The most common type of UI experienced by older CH residents is mixed UI, combining symptoms of overactive bladder (OAB -urgency, frequency, nocturia with or without urge UI) with stress UI^16^ however in the majority this is accompanied by functional losses associated with frailty^17^. No evidence of the effects of conservative interventions directly addressing mixed incontinence in CH populations is yet available^18^ and there is a dearth of published UK evidence of interventions to promote recovery of bladder continence in the CH context^19^ or for people living with dementia, even though this population is three times more likely to have UI or faecal incontinence (FI) as people of equivalent age and characteristics^20^. The absolute number of older adults with UI and associated level of dependency is predicted to increase rapidly with population ageing and the rise in prevalence of all forms of dementia, particularly in the oldest old, with consequent implications for future care provision^15^. The burden of UI in this population is significant and increasing^7^, yet evidence suggests that even intractable UI is amenable to interventions that may improve the older adults’ urinary function and quality of life^23^. Currently CHs use containment approaches rather than active treatment as the mainstay for managing UI, predominantly absorbent pads^41^. Other non-pharmacological options include voiding programmes, signage and environmental adaptations for people with dementia, bladder training and pelvic floor muscle training^24,25^ however evidence indicates these are rarely used, have limited effectiveness in the CH environment and are labour intensive^26^ which impacts on sustainability in the longer term. They also require a degree of cooperation, engagement and activity by the resident, which can be prohibitive for people with cognitive impairment^18,19^. Anti-muscarinic drugs may be used to reduce urge/OAB problems however these are associated with significant adverse effects in frail older people and avoided in those with dementia as they may also counteract the functional benefits of anticholinesterase inhibitors^27^.

Transcutaneous posterior tibial nerve stimulation (TPTNS) is a simple, non-invasive, safe and low-cost intervention with promising effectiveness, directly targeting urgency or mixed UI^1,22^. It uses a portable electrical nerve stimulation machine (TENS) to stimulate the posterior tibial nerve using surface electrodes placed adjacent to the medial malleolus. It does not require the resident to actively engage in order to receive the intervention, so is suitable for those who are frail and is comfortable to use^28^. It promotes dignified care as only access to the ankle is required and importantly it has the potential to treat UI in people with dementia.

Although the mechanism of action for TPTNS is not fully elucidated current evidence suggests it reduces involuntary detrusor contractions and increases cystometric capacity^28^ leading to a decreased sense of urgency, longer inter-void interval and greater sense of bladder control. By these means TPTNS offers potential for health gain in the CH population in terms of reduced urinary leakage, more appropriate use of the toilet and decreased use of continence products with associated improvements in quality of life. It has been shown to reduce UI in community-living older women^22^ and adults with neurogenic bladder dysfunction (including multiple sclerosis^29^, Parkinson’s^30^ and stroke^31^), however no studies have focused on treating UI in the CH population and only the applicants’ feasibility study indicates the safety, acceptability and potential effects of TPTNS in this context^1^.

With the increases in the older adult population and concomitant multi-morbidities including dementia^23^ together with the associated increase in UI, especially OAB/urge incontinence^7,16^ there is a pressing need to investigate interventions to treat UI to reduce the burden on CH residents and on care providers.

**1.2 Rationale for the trial**

A pilot randomised single-blind, placebo-controlled trial was previously completed to assess preliminary effects of a programme of TPTNS on lower urinary tract symptoms (LUTS) and number of episodes of UI in older care home residents and the feasibility of a full-scale randomised trial^1^. Thirty residents with LUTS and/or incontinence were recruited from seven residential care homes and 3 sheltered accommodation complexes. Participants received twelve 30-minute sessions of TPTNS or sham stimulation over a 6 week period and self-reported their LUTS at baseline and at the end of the 6 week intervention period using the American Urological Society Symptom Index (AUASI) and International Consultation on Incontinence Questionnaire Urinary Incontinence-Short Form (ICIQ UI-SF), and post-void residual urine (PVRU) volumes using portable bladder scanning. The results demonstrated AUASI scores improved, showing a median reduction of 7 (IQR -8 to -3) in the TPTNS group and a median increase in the sham stimulation group of 1 (IQR -1 to 4) (Mann-Whitney U 16.500, Z -3.742, P < .001). Total ICIQ UI-SF scores improved by a median of 2 (IQR -6 to 0) in the TPTNS group and 0 points (IQR-3 to 3) in the sham stimulation group (Mann-Whitney U 65.000, Z -1.508, P=.132). Analysis of change in PVRU volumes showed a difference in the mean reduction between the groups of 55.2 mL (95% confidence interval [CI] 0.5-110). This difference was significant (t = -2.215, df 11.338, P = .048). No adverse effects were reported by older adults or care staff and the pilot concluded that TPTNS is safe and acceptable with evidence of potential benefit for bladder dysfunction in older male and female residents of care homes^1^.

A systematic review of TPTNS for UI has been undertaken by our team using Cochrane methodology. Systematic searches of MEDLINE, EMBASE, CINAHL and the Cochrane Database of Systematic Reviews between 1980 and Feb 2016 identified ten randomised controlled trials (RCTs). Five trials used TPTNS in adults with UI only, four trials reported TPTNS in those with overactive bladder without UI and one trial included both UI and FI. A total of 472 participants were included, only 30 of whom were from a CH population. All studies reported improvements in bladder condition with TPTNS, in terms of symptom improvement and/or UI related quality of life, although no trial was definitive. A meta-analysis was possible (two trials) and found a mean reduction in the self-reported International Consultation on Incontinence Questionnaire- Urinary Incontinence (ICIQ-UI) short-form score of -3.79 (95%CI -5.82,-1.76), considered a clinically meaningful reduction^32^. There were no significant adverse events and TPTNS was consistently reported as safe. However, the studies in the meta-analysis were small (outcomes from 79 participants), had risk of bias associated methodological weaknesses and did not involve a fully CH population (or men in one of the two studies). The GRADE^62^ assessment of the certainty in the evidence for the meta-analysis gives a rating of ‘low quality’, meaning the true effect may be substantially different from the current estimate. Definitive evidence on the effectiveness of TPTNS is required before it can be recommended for routine practice in the CH context.

## 2. TRIAL AIM AND OBJECTIVES

**Aim:** The aim of the research is to determine the clinical effectiveness of a programme of transcutaneous posterior tibial nerve stimulation to treat urinary incontinence in care home residents and the associated costs and consequences.

**Objectives:**

1. To establish whether TPTNS is more effective than sham stimulation for reducing the volume of urinary incontinence at 6, 12 and 18 weeks, in older care home residents.

2. To investigate mediating factors that impact on the effectiveness of TPTNS in a mixed method, process evaluation involving fidelity, implementation support and qualitative components.

3. To undertake economic evaluation of TPTNS in care homes assessing the costs of providing the programme and presenting them alongside the key primary and secondary outcomes in a cost consequence analysis.

4. To explore in an interview study the experiences of TPTNS from the perspectives of:

• Care home residents

• Family carers

• Care home nurses and senior carers

• Care home managers

## 3. TRIAL DESIGN

The research comprises:

1. A pragmatic, multicentre, placebo controlled randomised parallel group trial to compare effectiveness of TPTNS (n=250) with sham stimulation (n=250) to reduce UI in CH residents. Results from an internal pilot with 100-140 residents will determine progression to full trial.

2. A longitudinal, mixed methods nested process evaluation investigating intervention fidelity and acceptability and qualitative components of the intervention and implementation support.

3. Economic evaluation of TPTNS compared with usual continence care pathway.

**3.1 Intervention being evaluated**

***Transcutaneous posterior tibial nerve stimulation*** (TPTNS) is a form of peripheral neuromodulation. The full 12 session programme (a total of 6 hours) is delivered in 30 minute sessions twice weekly over a 6-week period. The tibial nerve, which lies immediately posterior to the medial malleolus is stimulated electrically using a portable TENS machine and two surface electrodes. The cathode electrode is positioned behind the medial malleolus and the anode 10cm cephalad to it. Standardised stimulation parameters are applied of 10 Hz frequency, 200µs^-1^ pulse width in continuous mode and stimulation intensity (mA^-1^) is adjusted on a session-by-session basis according to individual resident comfort levels.

Although the exact mechanism of action has yet to be fully understood, TPTNS is believed to restore the balance between excitatory and inhibitory bladder functioning by modulating the signal traffic to and from the bladder through the sacral plexus^47^. It is hypothesised that stimulating afferent sacral nerves in the lower extremities increases the inhibitory stimuli to the efferent pelvic nerve, suppresses bladder afferent nerve activity, reduces detrusor contractility and increases bladder capacity^48^ and by these means TPTNS reduces the sensation of urgency and the frequency of micturition demanded, thus enabling improved bladder control. These mechanisms may also reduce the volume of urine retained in the bladder after voiding^1, 29^. For the CH population who wear absorbent pads because of mixed/urgency incontinence, TPTNS may reduce the sudden urge to urinate and frequency of voiding, allowing residents more time to reach the toilet, which in turn will enable more appropriate use of the toilet, generating respect and enhancing the person’s dignity.

As a potentially therapeutic modality TPTNS occupies a unique position in the CH care pathway for UI as it provides active treatment of the mixed/urge UI condition without requiring any active contribution by the resident, thus unusually, it is as likely to be of benefit to those with cognitive impairment as those without, and has been shown to be safe and not associated with any severe or limiting adverse effects.

***Sham stimulation*** comprises low intensity, sub-clinical stimulation of the lateral sub-malleolar area, positioned specifically on the lateral aspect to avoid the tibial nerve, which runs close to the skin surface behind the medial malleolus. The stimulation parameters are identical to the TPTNS stimulation other than the intensity of the current which will be set at 4mA, rather than adjusted individually as it is in the TPTNS intervention group. The current will be initially increased until the resident reports feeling some sensation following which the current will be reduced down to 4mA. All residents will be informed that they may not feel anything with this intervention and that this is quite normal.

Both intervention and sham groups will receive an electrical stimulation intervention comprising a total of 12 sessions of 30 minutes duration each, delivered twice weekly over 6 weeks. The intervention equipment and method of delivery are identical in everything but the intensity of electrical stimulation and the positioning of the surface electrodes. The electrical stimulation programme for all residents will be 10Hz frequency, 200μs pulse width, delivered in continuous stimulation mode. The electrical stimulators will be programmed to the set parameters and locked prior to individual use so that the only adjustable parameter will be the intensity of stimulation. The intervention will be delivered by CH registered nurses and senior carers who will receive specific training and support to undertake this role. Fidelity of delivery of the intervention and sham stimulation will be monitored.

No strict TPTNS/sham intervention timetable will be set and individual CHs will have flexibility around where, how and when they deliver the sessions, bearing in mind that they must occur twice weekly for 30 minutes each over a 6-week period. A proposed schedule for each home and resident will be agreed between the resident, registered nurse/senior carer and local PI at the point of treatment inception.

The allocated treatment (TPTNS or sham) will be offered to the resident a maximum of two times in any 24-hour period. If refused when first offered (verbally or by non-verbal behaviour) the treatment will be postponed for at least an hour and then offered one further time. Records of acceptance and refusals are documented in the resident’s treatment diary. Adherence to the TPTNS or sham stimulation programme is one of the progression criteria to full scale trial from the internal pilot. While aiming to complete a full 12-session programme over the 6-week intervention period contingency measures will be implemented if four or more sessions are refused by the resident or missed. Such measures will include approaching the resident at a later time (a maximum of two requests in any 24-hour period), different place or different day.

**3.2 Strategies to monitor adherence to intervention**

An individual resident stimulation diary is completed by the registered nurse/senior carer after delivering each session. It records date, time, intensity of electrical stimulation and any comments on the process of delivering the intervention. The locked stimulation machines automatically record the total stimulation time in use and the average stimulation intensity, thus an objective record of the stimulation programme provided to each resident is recorded, which is compared against the individually recorded stimulation diary. Electrode positioning by staff to deliver the intervention/sham stimulation will be recorded using a digital photograph taken by staff every two weeks during the intervention delivery time.

**3.3 Relevant concomitant care**

Implementing TPTNS or sham stimulation will not require alteration to current continence care pathways (including use of any medication such as antimuscarinics, alpha blockers, finasteride, vaginal oestrogen) and these will continue in line with CH policies for both trial arms.

**3.4 Internal pilot study**

An internal pilot will be undertaken that will focus on identifying feasibility of CH resident recruitment and retention throughout the 6-week trial intervention period and the success of strategies to support adherence and fidelity to the stimulation programme and completion of the primary outcome measure, the 24-hour pad weigh test (PWT).

We propose clear progression criteria for each below; these will be reviewed and finalised by the TSC in month -12 (June 2018). A decision about continuing to main trial will be taken during month 13 based on the pilot data collected between months 6 and 12. The data for each of the four criteria will be presented according to a traffic light system of **green**, to indicate *‘continue’*; **amber** to indicate the need to *‘implement contingency measures’*; **red** to indicate the need to *pause* the trial and investigate the possibility of discontinuing

**Pilot study success criteria**

***1.Recruitment:*** The overall target recruitment for the pilot study is at least 100 residents across the participating CHs. Number of CHs and recruitment rates will be monitored during the internal pilot and the following outcomes applied:

**Green:** recruitment of >90 residents. *Continue* as possible to recruit to time and target.

**Amber:** recruitment of 76-90 residents. *Continue but modify protocol* as may be possible to recruit to time and target with implementation of contingency plans (e.g. increased number of sites)**.**

**Red:** recruitment ≤75 residents or less. *Discuss discontinuation with TSC, DMEC, sponsor and funders.* We will review recruitment in each site on a monthly basis, both in the internal pilot and the main trial, to enable us to identify and address any issues as they arise.

***2. Adherence to stimulation:*** The target is for all residents to receive a minimum of two thirds of the electrical stimulation programme, which is at least 8 of the 12 sessions. These will be monitored by the stimulation diaries recorded by the administering CH staff as well as the data recorded by the electrical stimulators. The following outcomes will apply:

**Green:** >70% residents receive >8 stimulation sessions. *Continue without modification*.

**Amber:** 50-70% residents receive >8 stimulation sessions. *Continue but modify protocol.* (eg adjust timing and location of delivery to suit resident’s situation)

**Red:** <50% residents receive >8 stimulation sessions. *Discuss discontinuation* with *TSC,DMEC, sponsor and funders*.

***3. Completeness of 24hour pad weigh test (PWT):*** The target is a complete PWT at 6-weeks post-randomisation, for all participating residents. The following progression outcomes will apply

**Green:** >70% residents with complete 6-week PWT (no missing data). *Continue without modification*.

**Amber:** 50-70% residents with complete 6-week PWT. *Continue but modify protocol.* Implement contingency measures as planned (eg Repeat any incomplete PWTs; provide further staff education).

**Red:** <50% residents with complete 6-week PWT. *Discuss discontinuation with TSC, DMEC, sponsor and funders.*

***4. Fidelity to the allocated intervention (TPTNS or sham):*** The target is for all participating residents to receive the intervention protocol (duration and intensity of stimulation and correct ankle position) associated with the group to which they were allocated.

**Green:** >70% residents correctly receive >8 stimulation sessions. *Continue without modification*.

**Amber:** 50-70% residents correctly receive >8 stimulation sessions. *Continue but modify protocol.* Implement contingency measures as planned (eg alert CH staff to identified deficits and provide further education and support).

**Red:** <50% residents correctly receive >8 stimulation sessions. *Discuss discontinuation with TSC, DMEC, sponsor and funders.*

***Trial continuation decision***

Findings will be reviewed by the TSC and DMEC at 13 months. The trial will continue if the outcomes for all criteria are either green (**continue)** or amber (**continue but modify protocol)** and, if the latter, appropriate strategies can be identified to overcome the issues within an appropriate timeframe.

If the outcome is red **(Discuss discontinuation)**, we will discuss discontinuing with the TSC, DMEC, funder and sponsor. Options may include opening new sites if particular sites are performing poorly on all measures, or intensive re-education and support for the Implementation Support Facilitators.

# 4. TRIAL RECRUITMENT

**4.1 Trial population**

The trial will take place in >20 CHs (nursing or residential) for older adults in England and Scotland. Five hundred men and women resident in CHs who experience UI at least weekly, including those with cognitive impairment, will be recruited.

**4.2 Inclusion and exclusion criteria**

***Inclusion criteria:***

Care home residents:

- with self or staff reported UI of more than once/week
- who use the toilet or toilet aid for bladder evacuation with or without assistance
- who wear absorbent pads to contain UI.

***Exclusion criteria:***

- CH residents with an indwelling urinary catheter
- CH residents with symptomatic UTI
- CH residents with PVRU volume more than 300ml
- CH residents with a cardiac pacemaker
- CH residents with treated epilepsy
- CH residents with bilateral leg ulcers
- CH residents with pelvic cancer
- CH residents on the palliative care register
- Non-English speakers

**4.3 Identifying and approaching participants**

Processes for identifying eligibility and participant recruitment differ in England and Scotland according to relevant legislation on capacity to provide informed consent to participate.

*CH In England - Mental Capacity Act 2005:*

The local PI (senior clinical nurse or manager) in each CH will identify potentially eligible residents and complete a screening log. Study information will be provided to all potentially eligible residents. Where the local PI believes a resident’s capacity is in question, they will identify and provide the information to the resident’s personal consultee (usually a family member or friend). Where a personal consultee is not available a nominated consultee will be identified by the study team, in accordance with the MCA (2005). The local PI will seek agreement from the resident, if they have capacity or personal (or nominated) consultee where relevant, to speak to the RRA to gain information about the study.

*CH in Scotland -Adults with Incapacity (Scotland) Act 2000:*

The local PI (senior clinical nurse or manager) in each CH will identify potentially eligible residents and complete a screening log. Study information will be provided to all potentially eligible residents. Where there is evidence of incapacity (a resident has a certificate of incapacity) the local PI will identify and provide the study information to the resident’s welfare attorney (if one has been appointed) or their nearest relative. If there is no welfare attorney identified, or the resident does not have a relative who can be consulted, they will be considered ineligible to participate in the study. The local PI will seek agreement from the resident, if they have capacity or welfare attorney/nearest relative where relevant, to speak to the RRA to gain information about the study.

**4.4 Informed consent**

Procedures to seek and gain informed consent from eligible potential participants are agreed and confirmed by Research Ethics Committees with responsibility for reviewing applications for research with adults who lack capacity. The application for approval is made via the National Research Ethics Service (two separate submissions – England and Scotland). The research does not involve the NHS; therefore, management approval for CH sites to participate is sought via the individual CH group approval processes.

**Recruitment of residents**

All resident recruitment is undertaken by the RRAs for English and Scottish sites:

*CHs In England - Mental Capacity Act 2005:*

Following confirmation by the local PI of permission to approach the resident (and personal or nominated consultee) the RRA arranges to meet them to provide a full explanation of the study, ensure eligibility and seek consent to participate. Where the resident lacks capacity to consent the consultee provides advice on what they feel the person’s wishes would be if they had capacity. The consultee signs a declaration form if they believe the resident would choose to agree to participate. However, in accordance with the principles of the MCA (2005), ongoing consent (if resident has capacity) or assent is sought from the individual resident at every research contact and before any research activity is undertaken. Consultees may advise at any point that they believe the person’s wishes about participation have changed and they should therefore be withdrawn from study participation.

*CH in Scotland -Adults with Incapacity (Scotland) Act 2000:*

Following confirmation by the local PI of permission to approach the resident or their welfare attorney/nearest relative, the RRA arranges to meet them to provide a full explanation of the study, ensure eligibility and seek written consent to participate. The resident or their welfare attorney/nearest relative signs the consent form. However, in accordance with the principles of the Adults with Incapacity (Scotland) Act (2000) consent is not seen as an all or nothing entity and therefore ongoing verbal consent (if resident has capacity) or assent is sought from the individual resident at every research contact and before any research activity is undertaken. Welfare attorneys or nearest relatives may advise at any point that they withdraw consent for the resident to continue to participate.

**Recruitment of family carers**

Family members of participating residents who lack capacity to consent to participate in an interview are provided with study information by the local PI. Further explanation and the opportunity to ask questions about the study are offered to them by the RRA to enable an informed decision to be made about participation. Written consent to participate in the process evaluation interview is sought from the individual family carer prior to them taking part.

**Recruitment of staff**

Care home staff (RNs, SCs and managers) eligible to participate in the process evaluation will be provided with study information by the local PI. Further explanation and the opportunity to ask questions about the study are offered to them by the RRA to enable an informed decision to be made about participation. Written consent to participate in the process evaluation interviews is sought from the individual staff members prior to them taking part.

**4.5 Randomisation and allocation**

Following consent, baseline data will be collected. Only once all baseline data are recorded will remote randomisation be initiated. Eligible and consenting residents are randomised to one of the two groups (TPTNS or sham) using the proven 24-hour telephone Interactive Voice Response randomisation application or via the web-based application (and phone, if appropriate), both hosted by CHaRT. Information required to perform the randomisation is submitted by the RRA, who has obtained the consent, however to ensure the RRA is blinded to group allocation the information on the allocated group is delivered to the local PI in each CH by the ELECTRIC trial office, who receive the allocation information from CHaRT. The local PI records the allocated group in a separate file and informs the CH staff who will deliver the allocated intervention.

Randomisation will be computer allocated on a one to one basis in random permuted blocks of size two, four or six, with stratification by:

- Sex – male/female
- UI severity - mild (0-200ml/24 hrs); moderate (200-400ml /24 hrs); severe (400+ ml/24 hrs)
- Centre

**4.6 Administration arrangements post recruitment** (if applicable)

Following recruitment, the following administration arrangements will be undertaken:

- The Trial Office will notify the resident’s GP/medical practitioner in writing that the resident has joined the trial.
- The RRA will file a copy of the consent form in the resident’s care notes along with information about the trial.
- The RRA will file a copy of the GP/medical practitioner letter in the resident’s care notes.
- The RRA will enter trial data regarding the resident into the bespoke trial website.
- The RRA will maintain trial documentation at each site in their region (Scotland or England).
- The RRA will return a copy of the signed consent form to the Trial Office in Glasgow.

**4.7 Methods to protect against other sources of bias**

*Attrition*: The major anticipated source of attrition is resident death. Reasons for attrition are accurately recorded in each CH and therefore there should be minimal actual loss of residents to follow up at 18 weeks. Anticipated mortality rates have been accounted for in the sample size calculation and analysis plan.

*Use of sham stimulation as control*: To ensure the resident and their relatives are blind to the allocated intervention group a sham stimulation intervention rather than a no-treatment control is used.

*Contamination:* The risk that residents may receive the alternative intervention to which they were randomised will be addressed by the Implementation Support Facilitator, who will check digital photographs of electrode position taken by delivering nurses/senior carers on a bi-weekly basis and correct any delivery fidelity breach, including checking the stimulation diaries recorded after each session to ensure the information aligns with the allocated group. In addition, the electrical stimulator information downloads will be undertaken bi-weekly and will indicate any delivery protocol breaches, by comparing recorded parameters against the expected.

## 5. OUTCOME MEASURES

#### *5.1 Primary outcome measure*

Volume of UI leaked over a 24-hour period at 6 weeks post randomisation.

#### *5.2 Secondary outcome measures*

*Urinary outcomes*

Volume of UI leaked over a 24-hour period at 12 and 18 weeks post randomisation

Number of pads used in 24 hours at 6, 12 and 18 weeks post randomisation

Patient Perception of Bladder Condition (PPBC)^38^ at 6, 12 and 18 weeks post randomisation

Family Carer Perception of Bladder Condition (FC-PBC) at 6, 12 and 18 weeks post randomisation

Staff Perception of Bladder Condition (S-PBC) at 6, 12 and 18 weeks post randomisation

Minnesota Toileting Skills Questionnaire (MTSQ)^65^ at 6, 12 and 18 weeks post randomisation.

*Quality of Life outcomes*

Resident DEMQOL at 6 and 18 weeks post randomisation

Proxy DEMQOL at 6 and 18 weeks post randomisation^40^

*Economic outcomes*

Resource Use Questionnaire (RUQ) at 6 and 18 weeks post randomisation.

##

## 6. DATA COLLECTION AND PROCESSING

#### *6.1 Measuring outcomes*

Outcomes are measured at 6, 12 and 18 weeks post randomisation. The primary outcome is the volume of UI leaked in 24 hours at 6 weeks post randomisation, as measured by a 24-hour pad weight test (PWT)^37^. Pad weight tests at 12 and 18 weeks measure the sustainability of any effect. The test is based on the premise that 1g fluid weight = 1 ml urine and is thus an objective measure of urine leakage. The PWT involves the resident emptying their bladder, applying a clean, dry pad at an agreed set time and retaining all pads used between this time and 24 hours later. To maintain the moisture in the removed pads and prevent evaporation all collected pads are individually sealed in a small plastic bag and then placed in a larger re-sealable bag, which is weighed on site by the RRA after the 24-hour collection ends. The dry weight of the equivalent pads to those collected is deducted from the total weight to provide the 24-hour volume of UI leaked.

Secondary OCM include the *number of pads used in 24 hours*, which may be expected to reduce if TPTNS is effective in reducing volume of UI and will be reflected in the economic evaluation*.*

*Post void residual urine volume* (PVRU) is measured using a non-invasive portable ultrasound bladder scanner. Our pilot study conducted with CH residents^1^ suggested a potential mean decrease of 55ml in PVRU following a TPTNS programme compared to the sham stimulation group, it is thus worth investigating whether this was an artefact, or whether TPTNS impacts on urinary retention in the frail older adult population. Additionally, it is important to ensure that any effect of TPTNS in reducing bladder leakage is not as a result of an increase in retained urine volume.

The *Patient Perception of Bladder Condition* (PPBC) is a single question global patient reported outcome measure of perceived bladder condition with six possible responses ranging from ‘My bladder condition does not cause me any problems at all’ to ‘My bladder condition causes me many severe problems’. It has good construct validity and responsiveness to change^38^ and is recommended as a global outcome measure for UI^39^. It is used at each time point with residents. However, it has also been adapted in this study for use by family carers (FC-PBC) and CH staff (S-PBC) to offer a perspective on how they believe the resident feels about their bladder condition.

The *Minnesota Toileting Skills Questionnaire* (MTSQ) is a five question patient reported outcome measure of degree of difficulty on a scale of 0 to 4 completing five tasks involved in toileting. Scores range from 0 to 20, with higher scores indicating more difficulty. The MTSQ is a reliable and valid interviewer administered measure of toileting skills in physically frail older women^65^. It is completed at all timepoints by the resident and staff member.

Quality of life is measured using the *DEMQOL and DEMQOL-Proxy*^40^, valid and reliable measures of health-related quality of life in people with dementia. The DEMQOL-Proxy is completed by a single identified proxy for the resident. Both measures are completed at the primary outcome point (6 weeks post randomisation) and at the 18 week follow up assessment.

Economic evaluation is undertaken using routine data available in CHs as well as information from the Resource Use Questionnaire (RUQ) on which the RRA records at baseline the usual continence care pathway including details on usage of pads, medication which may affect continence, and (if appropriate) number of staff required to assist the resident to use the toilet. At the follow up time points, in combination with the 24 hour bladder diary, the RRAs use this questionnaire to update the continence care pathway. If residents have required any care from health professionals external to the CH as a result of their UI, this is also recorded on the RUQ.

Table 1 (below) summarises what outcomes are assessed at baseline, 6-week, 12-week and 18-week post randomisation assessments.

**Table 1**

|  | **Baseline** | **6-week** | **12-weeks** | **18weeks** | Data collector |
| --- | --- | --- | --- | --- | --- |
| 24 hour PWT | ● | ● | ● | ● | CH staff  RRA |
| Number of pads used | ● | ● | ● | ● | RRA |
| 24 hour bladder diary | ● | ● | ● | ● | CH staff |
| PVRU | ● | ● | ● | ● | RRA |
| PPBC | ● | ● | ● | ● | Resident and RRA |
| FC-PBC | ● | ● | ● | ● | Family member |
| S-PBC | ● | ● | ● | ● | SC/RN responsible for care provision |
| MTSQ | ● | ● | ● | ● | Resident and RRA |
| MTSQ | ● | ● | ● | ● | CH staff and RRA |
| DEMQOL | ● | ● |  | ● | Resident and RRA |
| DEMQOL-proxy | ● | ● |  | ● | Single, named proxy and RRA |
| Resource Use Questionnaire | ● | ● |  | ● | RRA |

**6.2 Baseline**

The RRA will complete the Case Report Form (CRF) for baseline data, which will record:

- Barthel Index (Mahoney & Barthel, 1965^60^)
- Mini Mental State Examination [MMSE] (Folstein et al, 1975^61^)
- Clinical Frailty Scale [CFS] (Rockwood, 2005^64^)
- Continence history and status, including Minnesota Toileting Skills Questionnaire [MTSQ] ^65^) and current treatment
- Falls and fractures, treated UTIs, emergency admissions to hospital in the previous 6 months
- Pressure ulcer risk and incidence in previous month
- 24 hour PWT
- Number of pads used in 24 hours
- PVRU volume measurements
- Resource Use Questionnaire
- Patient Perception of Bladder Condition
- Family Carer Perception of Bladder Condition
- Staff Perception of Bladder Condition
- DEMQOL
- DEMQOL-proxy
- 24-hour bladder diary information

**6.3 Follow-up**

***6 weeks post randomisation (Primary outcome point)*** (completion of TPTNS/sham programme):

The RRA will complete the CRF including:

- 24 hour PWT
- Number of pads used in 24 hours
- 24-hour bladder diary information
- MTSQ
- Continence status and current treatment
- PVRU volume measurements
- Resource Use Questionnaire
- Patient Perception of Bladder Condition
- Family Carer Perception of Bladder Condition
- Staff Perception of Bladder Condition
- DEMQOL
- DEMQOL-proxy

***12 weeks post randomisation:***

The RRA will complete the CRF including:

- 24 hour PWT
- Number of pads used in 24 hours
- 24-hour bladder diary information
- MTSQ
- Continence status and current treatment
- PVRU volume measurements
- Patient Perception of Bladder Condition
- Family Carer Perception of Bladder Condition
- Staff Perception of Bladder Condition

***18 weeks post randomisation:***

The RRA will complete the CRF including:

- 24 hour PWT
- Number of pads used in 24 hours
- 24-hour bladder diary information
- MTSQ
- Continence status and current treatment
- PVRU volume measurements
- Patient Perception of Bladder Condition
- Family Carer Perception of Bladder Condition
- Staff Perception of Bladder Condition
- DEMQOL
- DEMQOL-proxy
- Resource Use Questionnaire

**6.4 Capture of data from medical records**

Baseline data on medical condition is recorded from the residents medical and care records by the RRA after obtaining consent to participate, prior to randomisation. Data captured includes:

- Biographical information
- Medical history
- Current medication

**6.5 Change of Status/Withdrawal procedures**

Participants remain in the trial unless they (or their welfare attorney/nearest relative [Scotland]) choose to withdraw consent, or if their personal or nominated consultee (England) advises that they believe the person’s wishes about participation have changed or they are unable to continue for a clinical reason or if they die. All changes in status, with the exception of complete withdrawal of consent, mean the participant is still followed up for all trial outcomes wherever possible. All data collected up to the point of complete withdrawal is retained and used in the analysis.

If participants withdraw from the intervention or sham they (or their consultee [England] / welfare attorney/nearest relative [Scotland]), are asked to consider if they wish (the resident) to remain in the trial and be followed up as per trial schedule. Participants (or their consultee [England] / welfare attorney/nearest relative [Scotland]) who wish to withdraw from active trial follow-up are asked if they wish to allow routine follow-up data from CH records to be used for trial purposes.

If an individual CH wishes to withdraw from the study the permission of every participating resident (or their consultee [England] / welfare attorney/nearest relative [Scotland]) must be gained prior to CH withdrawal taking place.

**6.6 Data processing**

Paper based and electronic data entry will be used. RRAs enter locally collected data to the database in the CHs for screening and randomisation purposes. Paper based CRF data will be delivered securely to the Trial Office for data entry. Staff in the Trial Office work closely with local RRAs to ensure the data are as complete and accurate as possible.

## 7. SAFETY

**7.1 Standard definitions**

An **adverse event** (AE) is any untoward medical event affecting a clinical trial participant. Each initial AE is considered for severity, causality or expectedness and may be reclassified as a serious adverse event based on prevailing circumstances.

A **serious adverse event** (SAE), is any AE, that:

- results in death;
- is life threatening (i.e. the subject was at risk of death at the time of the event; it does not refer to an event which hypothetically might have caused death if it were more severe);
- requires hospitalisation or prolongation of existing hospitalisation;
- results in persistent or significant disability or incapacity;
- is otherwise considered medically significant by the investigator.

***7.2 Trial specific considerations***

In this trial, all AEs occurring during an electrical stimulation (treatment/sham) session, or while equipment is attached to the resident’s leg, or during data collection periods are recorded eg during questionnaire completion, interviews or 24-hour pad collections (see definitions below). Given the previous established safety profile of the TPTNS, serious related AEs are not anticipated in this low-risk trial. However, any serious related AEs that do occur will be recorded as such.

Hospitalisations for elective treatment of a pre-existing condition are not considered as an AE or SAE. Complications occurring during such hospitalisation are also not AEs or SAEs.

Falls, fractures, UTIs, emergency admissions to hospital in 6-month period prior to the resident’s participation in the ELECTRIC trial are recorded at study baseline. Falls, fractures and emergency admissions to hospital are not considered as related AEs/SAEs during the trial period unless they occur during a treatment session or during the measurement of study outcomes. Symptomatic UTIs treated with antimicrobials occurring at any time during the resident’s 18-week participation period, are considered to be AEs/SAEs.

**ELECTRIC specific treatment related expected adverse events:**

In this trial the following related minor AEs are potentially expected:

- Transient skin redness at electrodes sites
- Minor itch at electrode sites

There are no related serious AEs expected in this trial.

**7.3 Procedures for detecting, recording, evaluating & reporting AEs, SAEs**

**7.3.1 Detecting AEs and SAEs**

All AEs and SAEs meeting the criteria for recording within the ELECTRIC trial (see section 7.2) are recorded, from the time a participant consents to join the trial until the last trial follow-up. The RRA asks the participant/proxy (consultee [England], welfare attorney/nearest relative [Scotland]) or CH staff delivering the TPTNS/sham intervention about the occurrence of relevant AEs (i.e. those that meet the criteria for recording within the ELECTRIC trial) at each follow-up data collection point. AEs are also recorded in the stimulation diary.

**7.3.2 Recording AEs and SAEs**

When an AE/SAE meeting the criteria for recording within the ELECTRIC trial occurs, it is the responsibility of the local PI / RRA (or delegate) to review CH documentation related to the event. The local PI /RRA then records all relevant information about AEs in the relevant safety form. If an AE/SAE is recorded, the Trial Office liaises with the local PI /RRA to obtain further information if appropriate.

**7.3.3 Evaluating AEs and SAEs**

Seriousness, relatedness (causality), and expectedness is evaluated by a registered healthcare professional, including the local PI / RRA / CI

### *Assessment of Seriousness*

### The local PI / RRA / CI will make an assessment of seriousness as defined above.

### *Assessment of Relatedness (causality)*

The CI / RRA will make an assessment of whether the AE is likely to be related to research procedures according to the following definitions:

- **Related**: resulted from administration of TPTNS or sham stimulation or the outcomes data collection processes.
- **Unrelated**: where an event is not considered to have resulted from any of the research procedures.

Alternative causes such as natural history of any underlying disease, concomitant therapy, other risk factors and the temporal relationship of the event to the treatment are considered.

### *Assessment of Expectedness*

When assessing expectedness refer to the expected events (Section 7.2).

**7.3.4 Notification and reporting AEs and SAEs**

CH local PIs / RRAs are responsible for notifying the Trial Office of any AEs and SAEs meeting the criteria for recording within the ELECTRIC trial.

If an SAE form is submitted to the Trial Office, the Trial Manager is automatically notified. If, in the opinion of the local PI and/or the CI, the event is confirmed as being *serious* and *related* and *unexpected*, the CI or Trial Manager notifies the Sponsor within 24 hours of receiving the signed SAE notification. The Sponsor provides an assessment of the SAE. A Sponsor cannot downgrade an assessment from the PI or CI. Any disparity is resolved by further discussion between these parties. If all the required information is not available at the time of reporting, the local PI / RRA must ensure that any missing information is provided as soon as this becomes available. It should be indicated on the report that this information is follow-up information of a previously reported event.

**7.3.5 Regulatory reporting requirements**

The CI or delegate reports any SAEs that are related to any of the research procedures and unexpected to the appropriate REC (Scotland or England) within 15 days of the CI becoming aware of it using the HRA SAE form.

The CI is responsible for submitting annual reports to each REC on the anniversary of the approval. All related SAEs are summarised and reported to the Ethics Committee, the Funder, the Trial Steering Committee and the Data Monitoring Committee in their regular reports.

## 8. EMBEDDED QUALITATIVE WORK

#### *8.1 Overview*

The longitudinal process evaluation is undertaken concurrently with the RCT. The objectives are:

- To explore the experiences of the TPTNS intervention from the perspectives of residents, family carers and care home staff.
- To explore factors affecting intervention implementation in the care home context and optimisation for sustainability.

#### 8.2 Data collection

*Qualitative interviews:* Interviews are undertaken by a research assistant skilled in the application of qualitative methods and will explore experiences of TPTNS or sham stimulation and any perceived impact on continence status and quality of life from the perspective of the CH residents and their family carers. Attention will be given to understanding the intervention acceptability in the short and longer term, especially in comparison to other UI management strategies they may be familiar with and the identification of potential adherence moderating factors for future TPTNS delivery. All interviews will be digitally recorded and transcribed verbatim in preparation for analysis.

*Resident and/or family carer interviews*: Face to face semi-structured interviews will be undertaken with residents and/or family carers, either as individual interviews or dyads. A total of 20 interviews will be carried out at 6 weeks, on completion of the intervention. A maximum of 20 further interviews with different residents/carers will take place at the 12-week juncture. Purposive sampling of resident/carers for the qualitative interviews will be undertaken on the basis of maximum variability sampling^43^ with regard to gender, age, bladder symptoms, cognitive and functional status and resident or carer status. Three quarters of the interviews will involve residents who have received the TPTNS intervention or their families. A topic guide for the semi-structured approach will be developed to ensure all questions of interest are addressed. Fewer interviews will be conducted if data saturation is reached.

*CH nurses/senior carer interviews*: Focus group (or small group) interviews will be undertaken with CH nurses and senior carers involved in the direct delivery of the TPTNS/sham intervention. Attention will be paid to understanding the organisation of care, how management works with care staff, level of staff turnover in the previous six months and how continence care is organised within the routines of the care home. One focus group per CH or where for staffing reasons this is not possible to organise, 2-3 small group interviews, will be held during the month following the intervention completion. This will result in the equivalent of 20 focus group interviews involving 60-100 CH staff. Additionally, up to 20 individual interviews will be undertaken with nurses/senior carers delivering the intervention, to explore and elicit views which staff may be reluctant to share in a group interview.

*CH Managers:* Individual telephone interviews with CH managers (n = 20) will be completed at the end of each CH’s involvement with the study (6 months following site inception). The focus of these interviews will be to explore the CH culture and management values, perceived effects of the continence intervention at the organisational level, including any impact on culture and quality of care and any economic effects. Strategic considerations for implementation rollout and sustainability in the event that TPTNS is found to be effective will be identified and explored in depth.

***8.3 Data analysis***

For the three sets of qualitative interview data separate Framework analyses will be undertaken with the support of QSR NVivo (version 10) data management and analysis software. This method permits identification and cross-classification of variables directly from digital transcriptions. The analysis process consists of identifying key concepts and themes and mapping their range and diversity, followed by a process of interpretation where patterns of association are investigated and possible reasons for these explored. In achieving this all transcripts will be summarised, charted and coded for recurrent themes. Specific analytic intentions are associated with each of the three interview data sets:

*Residents/family carer interviews*: the framework will be developed to explore the elements of perceived impact and acceptability of TPTNS as a therapeutic intervention, by residents and family carers, in both the short-term and for the longer-term.

*CH staff:* The focus group and individual interview framework will highlight the experience of CH staff in developing their new skills set and the facilitators and challenges they experienced implementing them into routine practice. The elements of the COM-B model which formed the theoretical underpinning of the staff interview schedule will be key concepts in this framework.

*CH managers:* The focus of the framework for analysing the CH managers’ interviews will be the cultural, economic, strategic and quality impacts associated with participating in the trial and implications for implementation and sustainability at the organisational level.

The coherence, transparency and validity of the interpretations from these three different framework analyses will be assessed through regular iterative discussion between the RA with qualitative research experience and the study team members with qualitative expertise.

## 9. SAMPLE SIZE AND PROPOSED RECRUITMENT RATE

**9.1 Sample size**

A total of 500 residents will be required for this study, recruited across the two regions in accordance with allocation of RRA resources in that region. The worthwhile difference we believe the intervention will result in is a reduction of 200ml/24 hours. There is a lack of data available on the potential standard deviation of Pad Weight Tests^37^ (PWT) in CH residents; however, a small RCT^2^ reported results on this outcome. The SD from the trial was about 450ml, but given the small and selected sample included we estimated the upper 95% confidence interval around the SD which was approximately 570ml/24 hours, hence the standardised effect size of 0.35 (i.e. 200/570). To detect that difference with 90% power at the two-sided 5% alpha level we need primary outcome data on 344 participants. The limited data available suggested that the intra-class correlation (ICC) for any possible clustering effect was likely to be negligible; however, we have inflated this number to 500 to cover a conservative estimate of attrition to the primary outcome of about 30% due to death of CH residents and potential transfers to other CHs.

**9.2 Recruitment rates**

Large CHs with 100 or more residents and smaller CHs with a minimum of 25 residents will take part. The minimum population of residents will be 1700, although the expected number will be considerably higher. Approximately 70% will have UI and 10% will be in receipt of palliative care or do not use the toilet/toilet aid for elimination, thus there will be an estimated minimum total pool of 1071 eligible residents from whom we will recruit 500. Assuming similar recruitment rates to the HTA funded DCM-EPIC study undertaken in CHs of 60% of eligible CH residents, we would have at least 643 residents from whom to recruit our sample of 500. Recruitment will be undertaken over an 18-month period, commencing month 6 and will be completed by month 24. At least four new CH sites will be established every 3 months during the recruitment period, depending on CH size and location.

All trial and process evaluation data will be collected by month 30 (see Gantt chart: Figure 1).

## 10. STATISTICAL ANALYSIS

All analyses will be undertaken according to a previously agreed statistical analysis plan (SAP) and based on the intention-to-treat principle. The SAP will be agreed with the TSC, including the independent statistician, before any data analysis commences.

**10.1 Main effectiveness analysis:**

All baseline characteristics, follow-up measurements and safety data will be described using the appropriate descriptive summary measures: mean and SD for continuous and count outcomes or medians and inter-quartile range if required for skewed data, numbers and percentages for dichotomous or categorical outcomes.

The primary outcome, measured at 6 weeks post randomisation, will be analysed using linear regression correcting for baseline 24- hours PWT and other prognostic variables; all models will include a random effect for CH. The statistical analysis of the primary outcome will be by intention-to-treat (ITT); we will explore effects of compliance with treatment using causal models.  Potentially missing data will be handled using appropriate methods depending on the amount and pattern of missingness with sensitivity analysis to test assumptions.

Secondary outcomes will be analysed using a similar strategy employing generalised linear models suitable for the outcome. All treatment effects will be derived from these models and presented with 95% confidence intervals. All analysis will be performed and reported in accordance with the CONSORT statement and the ICH E9 ‘Statistical Principles in Clinical Trials’.

A single main analysis will be performed at the end of the trial when the 18 week follow up has been completed. An independent Data Monitoring and Ethics Committee will review confidential interim analyses of accumulating data, at its discretion but, at least annually.

**10.2 Planned subgroup analyses:**

Subgroup analyses will be carried out accordingly by:

- Gender
- UI severity
- Dependency in toilet use
- Cognitive status
- Falls status

Stricter levels of statistical significance (2P<0.01) will be sought, reflecting the exploratory nature of these analyses. Heterogeneity of treatment effects amongst subgroups will be tested for using the appropriate subgroup by treatment group interactions.

10.3 Process evaluation data analysis

Process evaluation data analysis will address the adherence to the stimulation programme by group, at time point 1 (6-weeks post-randomisation), the end of the stimulation programme. Characteristics of residents, and the stimulation programme they received will be described using appropriate summary measures and the proportion who received the therapeutic minimum > 8 stimulation sessions and the full 12 session programme presented. Overall fidelity to the allocated group will also be assessed and presented to illuminate resident elements of the outcome analysis including: total stimulation time, mean intensity of stimulation and accuracy of electrode position. Stimulation diaries will be analysed to identify data to inform our understanding of when, how and who delivers the electrical stimulation in practice.

11. ECONOMIC EVALUATION

The economic evaluation will compare the costs and outcomes of TPTNS compared with usual continence care pathways and present these in a cost consequence analysis.

**11.1 Collection of resources use and data**

Staff time required for training and the delivery of the intervention will be recorded by each CH, including the number of hours and the staff grade. This will be costed using the appropriate pay scales for each site. The costs of the trainer and the materials (TPTNS machines, TPTNS handbook and training DVD) will be based on the market rates for these items. A resource use questionnaire (RUQ) will be developed on which the RRA will record at baseline the usual continence care pathway including details on usage of pads and other equipment, medication which may affect continence level, and (if appropriate) number of staff required to assist resident to use the toilet. At the follow up time points, in combination with the 24-hour bladder diary, the RRA will use this questionnaire to update the continence care pathway. If residents have required any care from health professionals external to the CH as a result of their UI, this will also be recorded on the resource use questionnaire. Unit costs will be attached to the resources required using standard sources (including NHS Reference Costs, Unit Costs of Health and Social Care and British National Formulary^65-67^.

**11.2 Quality of Life**

Participant quality of life data is being collected as described in Section 6 on Outcomes.

**11.3 Analysis**

A cost consequence analysis presents the costs and outcomes in a disaggregated form (often called a ‘balance sheet’ approach). This approach is appropriate as there is more than one multidimensional outcome of importance which may not be captured via a Cost Effectiveness Analysis using the clinical primary outcome. It is possible to calculate utility values using the DEMQOL and DEMQOL–Proxy data. If the analysis of this data demonstrates a difference in QOL between the intervention and control arms, we will seek to calculate the utility values using the DEMQOL-U and calculate QALYs which could be used in a Cost Utility Analysis.

##

## 12. ORGANISATION: TRIAL MANAGEMENT AND OVERSIGHT ARRANGEMENTS

The Gantt chart (Figure 1) indicates when anticipated major trial events will occur, including recruitment, analyses and meetings. These time-related milestones will be used to enable close monitoring of progress.

## Figure 1 – ELECTRIC Gantt chart


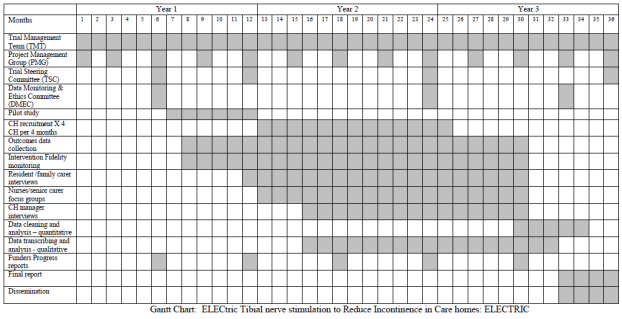


**12.1 Trial office in Glasgow**

The Trial Office is in the School of Health & Life Sciences, Glasgow Caledonian University and provides day to day support for the trial CH centres. The Trial Manager takes responsibility for the day to day transaction of trial activities, for example approvals, site set-up and training, oversight of recruitment and follow-up rates etc. The data co-ordinator provides clerical support to the trial, including organising all aspects of the data management eg postal questionnaires (mailing, tracking, and entering returned data using the trial web data entry portal). The Trial Manager and Data Coordinator will be supervised on a day to day basis by the Chief Investigator (JB) with support from the identified CHaRT representative. The qualitative researcher will be part of the Trial Office and will be supervised by ML, working closely with the CI. The Trial Office team meets formally at least monthly, (weekly during start up) during the course of the trial to ensure smooth running and trouble-shooting.

**12.2 Local organisation in CH sites**

The local PI and RRA in each site are responsible for all aspects of local organisation including identifying potential recruits, consenting, completing and maintaining appropriate documentation. The site agreement documents the full list of responsibilities for sites. Appropriate members of the local team are knowledgeable about the Protocol and will have appropriate Good Clinical Practice (GCP) training, if applicable. A trial-specific delegation log is prepared for each site, detailing the responsibilities of each member of staff working on the trial. The local team is also responsible for notifying SAEs to the Trial Office (see section 7).

**12.3 Project Management Group (PMG)**

The trial is supervised by its Project Management Group (PMG). This consists of the grant holders and representatives from the Trial Office and the CHaRT trial team. Observers are invited to attend at the discretion of the PMG. The PMG will meet/teleconference every three months on average, but monthly in the initial set-up stage.

**12.4 Trial Steering Committee (TSC)**

A Trial Steering Committee (TSC), with independent members, oversees the conduct and progress of the trial. The TSC Charter documents the terms of reference of the TSC, the template for reporting and the names and contact details of members of the TSC. This Charter is filed in the Trial Master File (TMF).

**12.5 Data Monitoring Committee (DMC)**

An independent Data Monitoring and Ethics Committee (DMEC) oversee the safety of subjects in the trial. The DMEC Charter documents the terms of reference of the DMEC and the names and contact details and is filed in the TMF. The Committee meets regularly to monitor the trial data and make recommendations as to any modifications that are required to be made to the protocol or the termination of all or part of the trial. CHaRT has adopted the DAMOCLES Charter for DMECs.

## 13. RESEARCH GOVERNANCE, DATA PROTECTION AND SPONSORSHIP

**13.1 Research Governance**

CHaRT is a fully registered Clinical Trials Unit with particular expertise in running multicentre RCTs. The ELECTRIC trial is run under the auspices of CHaRT based at HSRU, University of Aberdeen. This aids compliance with Research Governance and the principles of GCP, and provides support for aspects of trial administration, database support and statistical analyses. The CI ensures, through the TSC and Sponsor, that adequate systems are in place for monitoring the quality of the trial and providing appropriate expedited and routine reports, to a level appropriate to the risk assessment of the trial. The sponsors SOPs are followed and CHaRT SOPs, where appropriate.

**13.2 Data protection**

Data collected during the course of the research is kept strictly confidential and accessed only by members of the trial team, and may be looked at by individuals from the Sponsor organisation or CH sites where it is relevant to the participant taking part in this trial.

Participants are allocated an individual trial number. Participant’s details are stored on a secure database under the guidelines of the 2018 General Data Protection Regulation. The CHaRT senior IT manager (in collaboration with the CI) manages access rights to the data set. We anticipate that anonymised trial data may be shared with other researchers to enable international prospective meta-analyses.

**13.3 Sponsorship**

Glasgow Caledonian University is the sponsor for the trial.

## 14. ETHICS AND REGULATORY APPROVALS

The Yorkshire and The Humber Bradford Leeds Research Ethics Committee in England and the Scotland A Research Ethics Committee have reviewed this trial. The trial is conducted according to the principles of GCP provided by Research Governance Guidelines. Annual progress reports, end of trial declaration, and a final report are submitted to the Sponsor and the individual Research Ethics Committees in England and the Scotland within the timelines defined in the regulations.

**14.1 Protocol compliance and amendment**

The Investigators will conduct the trial in compliance with the protocol given favourable opinion by the Ethics Committee(s). Any amendment to the project is approved by the Sponsors and funder before application to REC, unless in the case of immediate safety measures when the Sponsor is notified as soon as possible. Any deviations from the Protocol will be fully documented using a breach report form.

## 15. QUALITY ASSURANCE

The trial is monitored to ensure that it is being conducted as per protocol, adhering to Research Governance, the principles of GCP, and all other appropriate regulations. The approach to, and extent of, monitoring is specified in the trial monitoring plan and is appropriate to the risk assessment of the trial. The ELECTRIC monitoring plan includes adherence to the Protocol Adherence Checklist which will be assessed during site monitoring visits undertaken by members of the Trial Office. Investigators and their host institutions are required to permit trial related monitoring and audits to take place by the Sponsor and/ or regulatory representatives, providing direct access to source data and documents as requested.

**15.1 Risk assessment**

An independent risk assessment has been carried out by the Sponsor.

## 16. FINANCE AND INSURANCE

The trial is funded by a grant awarded by the NIHR Health Technology Assessment programme (HTA Project: 15/130/73). The necessary trial insurance is provided by Glasgow Caledonian University.

## 17. END OF TRIAL

The end of follow-up for each participant is defined as the final data capture on that individual. The end of the trial is defined as the end of funding. The end of the trial will be reported to the Sponsor and REC within 90 days, or 15 days if the trial is terminated prematurely. If terminated prematurely, the Investigators will inform participants and ensure that the appropriate follow up is arranged for all involved, if appropriate. A summary report of the trial will be provided to the Sponsor and REC within one year of the end of the trial. An end of trial report is also issued to the funders at the end of funding.

## 18. DATA HANDLING, RECORD KEEPING AND ARCHIVING

Clinical data is entered into the database by the designated team members working in each CH site. Questionnaires returned to the trial office are entered there. Staff in the trial office work closely with local PIs and RRAs to ensure that the data are as complete and accurate as possible. Extensive range and consistency checks further enhance the quality of the data. Responsibilities for archiving are documented in the sponsor / site agreement. All essential data and documents (electronic and hard copy) are retained for a period of at least seven years after close of trial according to the funder requirements and relevant Sponsor and CHaRT archiving SOPs. Electronic data will be archived by the University of Aberdeen.

##

## 19. SATELLITE STUDIES

It is recognised that the value of the trial may be enhanced by smaller ancillary studies of specific aspects. Plans for these are discussed in advance with the PMG, and if appropriate with the TSC. Depending on the nature of the satellite study, the Sponsor may consider this to be a non-substantial or a substantial amendment to the REC approval for the ELECTRIC trial, or to require REC approval as a project in its own right. In such situations, the sponsor will be contacted for advice.

## 20. AUTHORSHIP AND PUBLICATION

To safeguard the integrity of the main trial, reports of explanatory or satellite studies will not be submitted for publication without prior arrangement from the PMG.

Once the main trial findings have been published, a lay summary of the findings will be sent to all involved in the trial.

Please refer to the Appendix 1 (authorship policy) for full details on authorship.

## REFERENCE LIST

## Booth J *et al.* A feasibility study of transcutaneous posterior tibial nerve stimulation for bladder and bowel dysfunction in elderly residents in residential care. *Journal of the American Medical Directors Association* 2013; 14, 270-274

## Vinsnes A *et al.* Effect of physical training on urinary incontinence: a randomized parallel group trial in nursing homes. *Clin. Interv Ageing* 7, 2012, 45–50

## Gale N *et al* (2013) Using the framework method for the analysis of qualitative data in multidisciplinary health research *BMC Medical Research Methodology* 13; 117

## Centre for Policy on Ageing - *A profile of residents in Bupa care homes: results from the 2012 Bupa census*. CPA, London, Nov 2012

## Saga S. What characteristics predispose to continence in nursing home residents?: A population based cross sectional study. *Neurourol Urodyn* 2015; 34: 362-367

## DuBeau C *et al*. Impact of urinary incontinence on quality of life in nursing home residents. *J Am Geriatr Soc* 2006;54:1325–33.

## Schumpf L *et al*. Urinary incontinence and its association with functional, physical and cognitive health among female nursing home residents in Switzerland. *BMC Geriatrics* 2017; 17:17 DOI 10.1186/s12877-017-0414-7

## Zarowitz B *et al.* Challenges in the pharmacological management of nursing home residents with overactive bladder or urinary incontinence*. JAGS* 2015, 63:2298-2307,

## Rait G *et al.* Prevalence of cognitive impairment: results from the MRC trial of assessment and management of older people in the community. *Age Ageing* 2005*,* 34(3), 242–248.

## Chiarelli P *et al*. Urinary incontinence is associated with an increase in falls: a systematic review. *Austral. J. Physiother.* 2009 55(2), 89–95

## Doughty D *et al*. Incontinence-Associated Dermatitis Consensus Statements, Evidence-Based Guidelines for Prevention and Treatment *J Wound Ostomy Continence Nurs*. 2012; 39(3):303-315.

## Yip S *et al*. The association between urinary and fecal incontinence and social isolation in older women. *Am J Obstet Gynecol*. 2013; 208:146.e1–7.

## de Vries H, Northington G, Bogner H. Urinary incontinence (UI) & new psychological distress among community dwelling older adults. *Arch Gerontol Geriatr* 2012; 55:49-54

## Barentsen J *et al*. Severity, not type is the main predictor of decreased quality of life in elderly women with UI: a population-based study as part of a RCT in primary care. *Health Qual Life Outcomes* 2012; 10 53.

## National Schedule of Reference Costs 2010/11 NHS Trusts and PCTs Combined Community and Outreach Nursing Services: Specialist Nursing

## International Consultation on Incontinence 5^th^ edition. Committee 11: *Incontinence in the frail elderly*. pp 1001-1099. 2013, ISBN : 978-9953-493-21-3

## Griebling T. Epidemiology, evaluation, and treatment of urinary incontinence in octogenarian women. *Curr Bladder Dysfunct Rep* 2014; 9:242–249

## DuBeau C. Beyond the bladder: management of UI in older women. *Clin Obstet Gynecol* 2007; 50:720–34.

## Wagg A *et al*. Urinary Incontinence in Frail Elderly Persons: Report From the 5th International Consultation on Incontinence *Neurourol. Urodynam*. 2015, 34:398–406,

## Stenzelius K *et al* The effect of conservative treatment of urinary incontinence among older and frail older people: a systematic review. *Age and Ageing* 2015; 0: 1–9

## Grant R *et al* First Diagnosis and Management of Incontinence in Older People with and without Dementia in Primary Care. *PLoS Med* 2013, 10(8): e1001505. doi:10.1371/journal.pmed.1001505

## Schreiner L *et al* Randomized trial of transcutaneous tibial nerve stimulation to treat urge urinary incontinence in older women. *Int Urogynecol J* 2010, 21:1065–1070

## Kinsella K, Wan H. *An aging world: 2008*. International Population Report *(*2009) .www.census.gov/prod/2009pubs/p95–09–01.pdf

## Wolff G, Kuchel G, Smith P. Overactive bladder in the vulnerable elderly. *Research and Reports in Urology* 2014:6 131–138

## Roe B *et al*. Systematic reviews of bladder training and voiding programmes in adults: a synopsis of findings from data analysis and outcomes using meta-study techniques. *J. Adv. Nurs.* 2007 57(1), 15–31.

## Schnelle J *et al*. Reduction of urinary incontinence in nursing homes: does it reduce or increase costs? *J Am Geriatr Soc*. 1988 Jan;36(1):34–39.

## Sink K *et al.* Dual use of bladder anticholinergics and cholinesterase inhibitors: Long-term functional and cognitive outcomes. *J Am Geriatr Soc* 2008;56:847–853

## Amarenco G *et al*. Urodynamic effect of acute transcutaneous posterior tibial nerve stimulation in overactive bladder. *The Journal of Urology*, 2003, 169:2210-2215

## De Seze N *et al*. Transcutaneous Posterior Tibial Nerve Stimulation for Treatment of Overactive Bladder Syndrome in MS: Results of a Multicenter Prospective Study *Neurourol. Urodyn*. 30:306–311, 2011.

## Perissinotto M A *et al*. Transcutaneous Tibial Nerve Stimulation in treatment of lower urinary tract symptoms & its impact on health related quality of life in patients with Parkinson’s *J Wound Ostomy Continence Nurs. 2015; 42(1):94-99.*

## Monteiro E *et al*. Electrical Stimulation of the Posterior Tibialis Nerve Improves Symptoms of Poststroke Neurogenic Overactive Bladder in Men: A Randomized Controlled Trial. *Urology* 84: 509-514, 2014

## Nystrom E *et al*. ICIQ Symptom and Quality of Life Instruments Measure Clinically Relevant Improvements in Women With Stress Urinary Incontinence. *Neurourology and Urodynamics* 34:747–751, 2015

## Stetler CB *et al*: Role of 'external facilitation' in implementation of research findings: a qualitative evaluation of facilitation experiences in the Veterans Health Administration. *Implementation Science*. 2006, 1: 1-15. 10.1186/1748-5908-1-1.

## Michie *et al*. The behaviour change wheel: A new method for characterising and designing behaviour change interventions. *Implementation Science* 2011 6:42

## Harvey G *et al*. Getting evidence into practice; the role and function of facilitation*. J Adv Nurs*. 2002, 37 (6): 577-588. 10.1046/j.1365-2648.2002.02126.x.

## Cheater FM *et al*. Cluster randomised controlled trial of the effectiveness of audit and feedback and educational outreach on improving nursing practice and patient outcomes [for urinary incontinence]. *Medical Care*. 2006, 44 (6): 542-551.

## Dylewski DA, *et al*. A statistical comparison of pad numbers versus pad weights in the quantification of urinary incontinence. *Neurourol Urodyn* 2007; 26: 3–7

## Coyne KS *et al.* The validation of the patient perception of bladder condition (PPBC): a single-item global measure for patients with overactive bladder. *Eur Urol*. 2006 Jun;49(6):1079-86.

## Sloan JA, Aaronson N, Cappelleri JC. Assessing the clinical significance of single items relative to summated scores. *Mayo Clin Proc* 2002; 77:479-487.21

## Smith SC, Lamping DL, Banerjee S. Measurement of health-related quality of life for people with dementia: development of a new instrument (DEMQOL) and an evaluation of current methodology. *Health Technol Assess.* 2005 Mar;9(10):1-93.

## Omli R *et al*. Pad per day usage, urinary incontinence and urinary tract infections in nursing home residents. *Age Ageing* 2010, 39, 549-554

## Hasson H (2010) Systematic evaluation of implementation fidelity of complex interventions in health and social care *Implementation Science* 5, 67

## Patton M: *Qualitative Evaluation and Research Methods.* 1990, Newbury Park, CA: Sage

## Bellette P *et al*. Posterior tibial nerve stimulation in the management of overactive bladder: A prospective and controlled study. *Actas Urologicas Espanolas* 2009;33:58e63

## Barry MJ, Fowler FJ, O’Leary M. The American Urological Association Symptom Index for Benign Prostatic Hyperplasia. *J Urol* 1992;148:1549e1557.

## Avery K, *et al*. ICIQ: A brief and robust measure for evaluating the symptoms and impact of urinary incontinence. *Neurourol Urodyn* 2004;23:322e330.

## Al-Shaiji TF, Banakhar M, Hassouna MM. Pelvic Electrical Neuromodulation for the Treatment of Overactive Bladder Symptoms. *Advances in Urology*, 2011, Article ID 757454, 7 pages, doi:10.1155/2011/757454

## Choudhary M, van Mastrigt R,van Asselt E. Effect of tibial nerve stimulation on bladder afferent nerve activity in a rat detrusor overactivity model. *International Journal of Urology* (2015) doi: 10.1111/iju.13033

## Nishtala PS, Salahudeen SM, Hilmer SN. Anticholinergics: theoretical and clinical overview. *Expert Opinion On Drug Safety* Vol. 15 , Iss. 6, 2016

## Wagg A. The Cognitive Burden of Anticholinergics in the Elderly–Implications for the Treatment of Overactive Bladder. *European Urological Review*. 2012;7(1):42–9

## Svihra J, Kurca E, Luptak J. Neuromodulative treatment of overactive bladder: Non-invasive tibial nerve stimulation. *Bratisl Lek Listy* 2002;103:480e483.

## Chen G, Liao L, Li Y The possible role of percutaneous tibial nerve stimulation using adhesive skin surface electrodes in patients with neurogenic detrusor overactivity secondary to spinal cord injury *Int Urol Nephrol* (2015) 47:451–455

## Manrıquez V *et al* Transcutaneous posterior tibial nerve stimulation versus extended release oxybutynin in overactive bladder patients. A prospective randomized trial. *Eur Journal of Obstetrics & Gynecology and Reproductive Biology* 196 (2016) 6–10

## Treweek S, Zwarenstein Making trials matter: pragmatic and explanatory trials and the problem of applicability *Trials* 2009, 10:37 doi:10.1186/1745-6215-10-37

## Medical Research Council. *Medical research involving adults who cannot consent*. London: Medical Research Council 2007.

## Dobson C. *Conducting research with people not having the capacity to consent to their participation. A practical guide for researchers*. Leicester: British Psychological Society 2008.

## Mental Capacity Act, (2005).

## Adults with Incapacity (Scotland) Act 2000

## Guest G, Bunce A, Johnson L (2006). How many interviews are enough? An experiment with data saturation and variability. *Field Methods* 18 (1); 59-82

## Mahoney FI, Barthel D. “Functional evaluation: the Barthel Index” *Maryland State Medical Journal* 1965;14:56-61.

## Folstein, M., Folstein, S.E., McHugh, P.R. (1975). “Mini-Mental State” a Practical Method for Grading the Cognitive State of Patients for the Clinician. *Journal of Psychiatric Research*, 12(3); 189-198.

## Guyatt GH *et al*. GRADE: an emerging consensus on rating quality of evidence and strength of recommendations. *BMJ* 2008; 336: 924–6.

## Krhut J et al. 2014. Pad weight testing in the evaluation of urinary incontinence. *Neuro Urol Urodynam* 33:507-510

## Rockwood et al. A global clinical measure of fitness and frailty in elderly people. *CMAJ* 2005;173:489-495

## Talley KM *et al.* Reliability and validity of two measures of toileting skills in frail older women without dementia*. J Gerontol Nurs* 2016;3:1–5.

## Curtis L: *Unit costs of health and social care.* Canterbury: University of Kent; 2015

## Department of Health. *NHS Reference Costs 2014-2015J*

## *J*oint Formulary Committee: *British National Formulary*. London: BMJ Group and Pharmaceutical Press

**APPENDICES**

## Appendix 1: AUTHORSHIP POLICY FOR ELECTRIC STUDY

1. **DEFINING AUTHORSHIP**

Authorship of published or presented papers is based on the following criteria^1^:

1. Substantial contributions to the conception or design of the work; or the acquisition, analysis, or interpretation of data for the work; AND
2. Drafting the work or revising it critically for important intellectual content; AND
3. Final approval of the version to be published; AND
4. Agreement to be accountable for all aspects of the work in ensuring that questions related to the accuracy or integrity of any part of the work are appropriately investigated and resolved.
5. **PRINCIPLES OF AUTHORSHIP**

The following principles of authorship have been derived from editorial publications from leading journals^2,3^ and are in accordance with the rules of the International Committee of Medical Journal Editors (ICMJE)^1^.

- All contributors must fulfil the criteria detailed in section 1: DEFINING AUTHORSHIP in order to qualify for authorship.
- Contributors who meet fewer than all four of the criteria for authorship listed above should not be listed as authors, but they should be acknowledged. For example, participation solely in the acquisition of funding, collection of data or technical editing, language editing or proof reading the article is insufficient by itself to justify authorship^1^. Those persons may be acknowledged and their contribution described. See section 3: ACKNOWLEDGEMENTS.
- Where possible studies should be published using all the named contributors who qualify for authorship in the byline i.e. Jane Doe, John Doe, John Smith and Ann Other. However, there may be situations where this is not possible, for example if the journal limits the number of authors. In such circumstance, group authorship may be appropriate using bylines similar to “The ELECTRIC trial group”. The article should carry a footnote of the names of the people (and their institutions) represented by the corporate title.

1. **Determining authorship**

Authorship criteria are intended to preserve the status of authorship for those who deserve credit and can take responsibility for the work. The criteria are not intended for use as a means to disqualify colleagues from authorship who otherwise meet authorship criteria by denying them the opportunity to meet criterion numbers (ii) or (iii). Therefore, all individuals who meet the first criterion should have the opportunity to participate in the review, drafting, and final approval of the manuscript^1^.

Tentative decisions on authorship should be made as early as possible^3^. These should be justified to, and agreed by, the Project Management Group. Any difficulties or disagreements will be resolved by the Trial Steering Committee (TSC).

1. **Ordering of authors**

The following rules may help with the ordering of authors, particularly for publications with individual authorship:

The person who has taken the lead in writing may be the first author.

The senior author may wish to be the last named author.

1. Those who have made a major contribution to analysis or writing (i.e. have done more than commenting in detail on successive drafts) may follow the first author immediately; where there is a clear difference in the size of these contributions, this should be reflected in the order of these authors.
2. All others who fulfil the four authorship criteria described in Section 1: DEFINING AUTHORSHIP may complete the list in alphabetical order of their surnames.
3. **ACKNOWLEDGEMENTS**

In all published papers, posters etc the list of authors must end with term "on behalf of the ELECTRIC trial consortium". In case a journal deems this acknowledgement unacceptable, the phrase "within the framework of the ELECTRIC trial" shall be included in the acknowledgements section of the article or presentation. All those in the consortium who do not fulfil the criteria for authorship, should then be acknowledged by name, in the ‘Acknowledgements’ section. A full list of consortium members should be obtained from the ELECTRIC trial manager. Because acknowledgment may imply endorsement by acknowledged individuals of a study’s data and conclusions, authors are advised to obtain written permission to be acknowledged from all acknowledged individuals^1^.

All publications and reports of work arising from the ELECTRIC trial must adhere to the NIHR branding guidance found in the ‘Project outputs guidelines’

1. **DISCLAIMERS**

Authors should ensure they include the study funder’s disclaimer: refer to the funders website for details. Be aware that other disclaimers may also be required.

1. **QUALITY ASSURANCE**

Ensuring quality assurance is essential to the good name of the trial group. All reports of work arising from the ELECTRIC trial, including conference abstracts, should be peer reviewed by the Project Management Group. The Project Management Group will be responsible for decisions about submission following internal peer review. Submission may be delayed or vetoed if there are serious concerns about the scientific quality of the report. If individual members of the group are dissatisfied by decisions, the matter may be referred to the TSC.

It is hoped that the adoption and dissemination of this policy will prevent disputes that cannot be resolved by informal discussion. However, any member of the study team with a concern about authorship should discuss it with the relevant Chief Investigator, TSC, Line Manager or Programme Director as appropriate.

**References**

1. Recommendations for the Conduct, Reporting, Editing, and Publication of Scholarly Work in Medical Journals. Developed by members of the ICMJE over the period 2011 to 2013. ([www.icmje.org/#authors](http://www.icmje.org/#authors))
2. Huth EJ (1986). Guidelines on authorship of medical papers. Annals of Internal Medicine, **104**, 269-274.
3. Glass RM (1992). New information for authors and readers. Group authorship, acknowledgements and rejected manuscripts. Journal of the American Medical Association, **268**, 99.

<<Insert here the current version of the ChaRT Authorship Policy template held on Q-Pulse.>>

**Appendix 2 - Study flowchart**

ELECTRIC Study flowchart

| **Randomised Controlled Trial** | | **Process evaluation** | | |
| --- | --- | --- | --- | --- |
| >20 Care homes  Care home residents (n = 500)  Eligibility confirmed  Consent | | **Intervention delivery fidelity**  Stimulation delivery diaries completed by  CH nurses / senior carers  Stimulation time records downloaded from locked stimulators bi- weekly | **Research process fidelity**  Internal pilot –  first 100-140 participants:  24 Hour PWT  Record at 6, 12, 18 weeks  Number pads used/24 hours  PVRU  PPBC  FC-PBC  S-PBC  MTSQ  Record at 6, 12, 18 weeks  DEMQOL, DEMQOL proxy  Record at 6 & 18 weeks  Site visits to all centres  Protocol adherence checklist | **Qualitative evaluation**  Individual interviews with purposive sample of CH residents and/or family carers at 6 weeks (n = 20)  Individual interviews with purposive sample of CH residents and/or family carers at 12 weeks (n <20)  Focus group interviews with CH nurses and senior carers who deliver stimulation:  1 per care home = 20 focus group interviews  Individual interviews with CH nurses/senior carers who deliver stimulation(n <20)  Individual interview with care home manager (n= 20) |
| Baseline: T0  24 hour PWT  Number pads used/ 24 hours  PVRU, PPBC, FC-PBC, S-PBC  DEMQOL, DEMQOL proxy,  MTSQ, RUQ | |
| Randomised | |
| TPTNS intervention  n= 250  Delivered by CH nurses/senior carers over 6 weeks | Sham stimulation  n=250  Delivered by CH nurses/senior carers over 6 weeks |
| 6 weeks: T1  24 hour PWT  Number pads used/ 24 hours  PVRU, PPBC, FC-PBC, S-PBC  DEMQOL, DEMQOL proxy  MTSQ, RUQ | |
| 12 weeks: T2  24 hour PWT  Number pads used/ 24 hours  PVRU,  PPBC, FC-PBC, S-PBC, MTSQ | |
| 18 weeks: T3  24 hour PWT  Number pads used/ 24 hours  PVRU, PPBC, FC-PBC, S-PBC  DEMQOL, DEMQOL proxy  MTSQ, RUQ | |

Key – 24 Hour Pad Weight Test (PWT); Post-void residual urine volume (PVRU); Patient Perception of Bladder Condition (PPBC); Family Carer Perception of Resident Bladder Condition (FC-PBC); Staff Perception of Resident Bladder Condition (S-PBC); Dementia Quality of Life (DEMQOL); Minnesota Toileting Skills Questionnaire (MTSQ); Resource Use Questionnaire (RUQ)

Resident flowchart


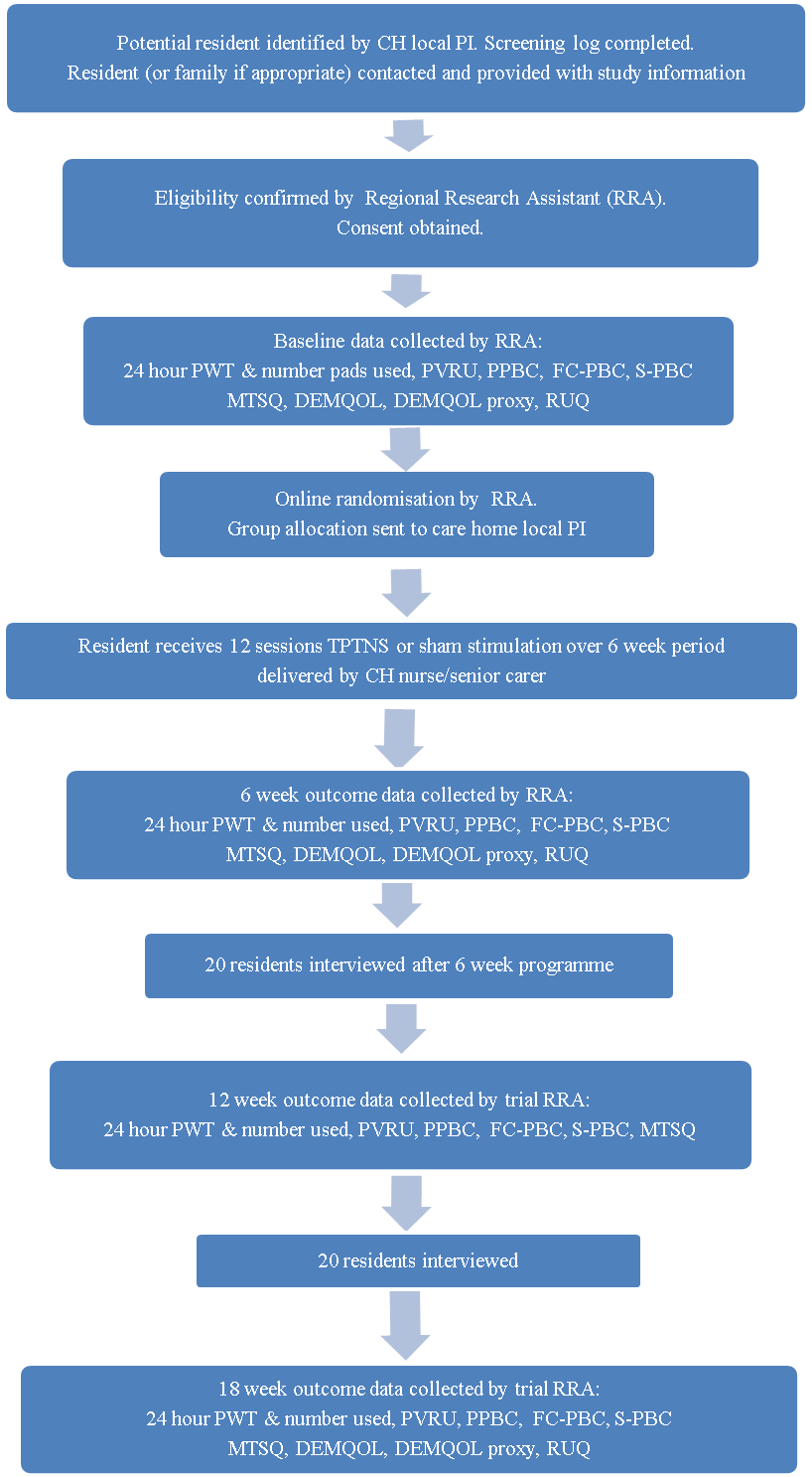

Supplement: Supplementary file 1 — Additional file 1. Current version of trial protocol. [file 13063_2019_3723_MOESM1_ESM.doc]
