# Supplementary material for: ELECtric Tibial nerve stimulation to Reduce Incontinence in Care homes: protocol for the ELECTRIC randomised trial
Source: Trials. 2019 Dec 16;20:723. doi: 10.1186/s13063-019-3723-7 (PMC6915984; doi:10.1186/s13063-019-3723-7)
Supplement: Supplementary file 2 — Additional file 2. Resource Use Questionnaire designed for the ELECTRIC trial. [file 13063_2019_3723_MOESM2_ESM.docx]

# ELECTRIC Trial: Resource Use Questionnaire (RUQ)

## Medication prescribed for incontinence

| Name of medication (generic name preferred) | Dose per day | Ongoing medication? (Y/N) | If No, indicate how many days medication prescribed |
| --- | --- | --- | --- |
|  |  |  |  |
|  |  |  |  |
|  |  |  |  |
|  |  |  |  |
|  |  |  |  |
|  |  |  |  |
|  |  |  |  |
|  |  |  |  |

## Appointments with health service staff for incontinence problems (in past 6 weeks)

| Health Service staff | Number of appointments | In care home (Y/N) |
| --- | --- | --- |
| GP |  |  |
| Practice Nurse |  |  |
| District Nurse |  |  |
| Physiotherapist |  |  |
| Occupational Therapist |  |  |
| Continence Service |  |  |
|  |  |  |
|  |  |  |

## Is assistance required from care home staff to attend toilet? Yes /No  ________________

1. **If yes, please indicate how many staff are required per visit :____________________**
2. **If yes, please indicate how many visits (on average) are required per day:___________**

**(see bladder diary)**

## Has any special equipment been provided as a result of participant incontinence (eg. Sheets, hoist, commode) DO NOT INCLUDE ABSORBENT PADS

| Item | Used on a daily basis? |
| --- | --- |
|  |  |
|  |  |
|  |  |
|  |  |

TO BE COMPLETED BY THE TRIAL OFFICE:


## Time taken by care home staff to complete stimulations (taken from stimulation diary)

| **Staff grade *of Neurotrac administrator** | **Average time taken to set up stimulation over 12 week period (minutes)**** |
| --- | --- |
|  |  |
|  |  |
|  |  |
|  |  |
|  |  |
|  |  |
|  |  |

*Trial Office should examine stimulation diary and, using initials of staff administering the stimulation, contact the local PI to ascertain the staff grade

**The total time taken to SET UP each stimulation should be calculated FOR EACH STAFF GRADE and divided by the number of stimulations administered by staff of that grade.
